# Esketamine exerts a protective effect against skeletal muscle injury induced by hindlimb ischemia-reperfusion in mice by regulating autophagy

**DOI:** 10.1371/journal.pone.0358125

**Published:** 2026-09-15

**Authors:** Jingwang Liu, Jiaxin Liu, Tianyi He, Penghui Zhang, Nan Zhao, Maozheng Wei, Yuxin Guan, Peng Liu, Shuang Zhao, Xiuli Wang

**Affiliations:** 1 Department of Anesthesiology, The Third Hospital of Hebei Medical University, Shijiazhuang, Hebei, China; 2 Department of Physiology, Hebei Medical University, Shijiazhuang, Hebei, China; University of Minnesota Medical School, UNITED STATES OF AMERICA

## Abstract

Tourniquet-associated lower limb ischemia-reperfusion can induce skeletal muscle inflammation, edema, and early functional impairment, in which autophagy dysregulation may be involved. The present study was designed to investigate the effects of esketamine (ESK) on skeletal muscle injury following lower limb ischemia-reperfusion in mice and its relationship with autophagy regulation. A mouse model of left hindlimb ischemia for 3 hours followed by 24 hours of reperfusion was established, and changes in hindlimb perfusion, functional outcomes, tissue injury, inflammatory response, and autophagy-related markers were examined after intervention with ESK alone or in combination with chloroquine phosphate (CQ). The results showed that ESK attenuated the abnormally elevated perfusion after reperfusion, improved motor performance and partial muscle contractile function, yet exerted limited effects on mechanical pain threshold and nerve stimulation-induced muscle contraction. Concurrently, ESK alleviated histological damage, edema, and sarcolemmal integrity disruption in the gastrocnemius muscle, reduced serum Tumor Necrosis Factor-alpha and Interleukin-6 levels, and downregulated the expression of several inflammation-related genes. With respect to autophagy, ESK increased the microtubule-associated protein 1 light chain 3 II/I ratio while decreasing sequestosome 1 levels and the phosphorylated mechanistic target of rapamycin/total mechanistic target of rapamycin ratio; these protective effects were partially abrogated by CQ. Collectively, these findings indicate that ESK mitigates acute-phase skeletal muscle injury and improves selected early functional outcomes after lower limb ischemia-reperfusion in mice, and that its protective actions may be associated with suppression of inflammation and modulation of autophagy.

## Background

In orthopedic surgery, tourniquets are widely used because they effectively reduce intraoperative bleeding and provide a clear surgical field [1]. However, during tourniquet application, patients often develop the so-called “tourniquet response,” characterized by progressive pain and sympathetic excitation caused by sustained limb ischemia, which manifests as increases in blood pressure and heart rate. This poses considerable challenges for intraoperative anesthetic management, requiring anesthesiologists to repeatedly balance adequate analgesia and sedation, hemodynamic stability, and the avoidance of drug overdose [2,3]. Meanwhile, the adverse effects induced by tourniquet use do not completely cease at the end of surgery. After tourniquet release and restoration of blood flow, the ischemia-reperfusion (I/R) process can further trigger excessive generation of reactive oxygen species and inflammatory cascade responses, resulting in injury to multiple tissues, including muscle, nerve, and vasculature [4]. Clinically, this is manifested as early postoperative lower-limb pain, weakness, restricted mobility, and delayed functional recovery, all of which markedly reduce patient comfort and willingness to ambulate, thereby slowing postoperative rehabilitation [4,5]. This has become one of the important factors limiting the broader implementation of the Enhanced Recovery After Surgery (ERAS) concept in orthopedics.

I/R injury is essentially a multifaceted form of tissue trauma. Previous studies have shown that it can simultaneously damage the muscular, neural, and vascular systems, leading to disruption of myofiber architecture, impaired nerve conduction, and microcirculatory perfusion deficits. The underlying mechanisms involve complex pathological processes, including enhanced oxidative stress, mitochondrial dysfunction, elevated inflammatory cytokines, local edema, and apoptosis [6,7]. As an important metabolic process for maintaining neuronal and muscular cellular homeostasis, autophagy plays a “double-edged sword” role in I/R injury: moderate autophagy can remove damaged proteins and mitochondria, thereby alleviating oxidative stress and neuroinflammation; however, insufficient or excessive autophagy may both aggravate cellular injury [8,9]. Therefore, whether modulation of autophagy can be used to ameliorate I/R injury has become a current focus of research.

Esketamine (ESK) is a highly selective N-methyl-D-aspartate (NMDA) receptor antagonist that is widely used in perioperative anesthetic management because of its prominent sedative and analgesic effects [10,11]. In orthopedic surgery, particularly in procedures requiring prolonged tourniquet application, ESK can effectively alleviate intraoperative pain and sympathetic excitation caused by the tourniquet response, and has therefore become one of the commonly used agents for optimizing anesthetic regimens in orthopedics [2,12]. Notably, beyond its analgesic properties, accumulating evidence in recent years suggests that ESK may exert protective effects under pathological conditions such as I/R and oxidative stress by regulating autophagy and controlling inflammation [13,14]. However, under the condition of hindlimb I/R, systematic evidence is still lacking as to whether ESK can likewise confer protection. Therefore, the present study aimed to establish a mouse hindlimb I/R model to evaluate the effects of ESK on hindlimb I/R-related functional outcomes and tissue injury, thereby providing a new theoretical basis and experimental evidence for optimizing anesthetic strategies in orthopedic surgery and promoting rapid postoperative recovery.

## Materials and methods

### Animals

This experimental protocol was approved by the Animal Care and Use Committee of Hebei Medical University (Approval No. IACUC-Hebmu-2025129; approval date: December 17, 2025). The animals used in this study were specific pathogen-free male C57BL/6 mice (n = 70), aged 8–10 weeks and weighing 20–25 g, supplied by Hebei Zizhen Biotechnology Co., Ltd. The mice were housed in an animal facility under a 12 hours (h) light/12 h dark cycle, at an ambient temperature of 20–24°C and a relative humidity of 40%–60%, with ad libitum access to food and water. All mice were acclimated for 7 days before the start of the experiment. General health status was assessed preoperatively, and only healthy mice without obvious signs of disease or behavioral abnormalities were included in the subsequent experiments. Mice with body weights outside the specified range or with unsuccessful model establishment were excluded. All animals meeting the inclusion criteria were included in the analysis, with no additional exclusions. All animal procedures were conducted in strict accordance with the institutional guidelines for animal experimentation, and the study was reported in compliance with the ARRIVE 2.0 guidelines.

In this study, humane endpoints were predefined for all animals. If an animal exhibited severe pain or distress, euthanasia was performed promptly. Animal health status and behavior were monitored throughout the experiment, with continuous observation during anesthesia and recovery, and twice-daily monitoring after surgery to ensure animal welfare. The specific humane endpoints included inability to eat or drink; severe lethargy with no response to stimulation; severe dyspnea; progressive marked swelling, ulceration, or necrosis of the affected hindlimb; evidence of self-mutilation tendency or obvious tissue loss; or a body weight loss of more than 20% relative to baseline. Once any of these endpoint criteria were met, immediate euthanasia was carried out. In this study, no animal died before meeting the euthanasia criteria.

The experimental duration for each animal consisted of 3 h of hindlimb ischemia followed by 24 h of reperfusion. At the end of the 24 h reperfusion period, all animals were euthanized at the predetermined experimental endpoint after completion of the corresponding experimental procedures. A total of 70 animals were used, and all animals ultimately underwent humane euthanasia. All surgeries and procedures were performed under isoflurane anesthesia (1.5-3%), and measures such as the use of heating pads were implemented to minimize pain and stress as much as possible. Euthanasia was performed by carbon dioxide inhalation. All experiments were conducted by trained researchers in accordance with relevant ethical and technical standards.

### Experimental design and animal grouping

A total of 70 mice were included in this study. The main experimental cohort consisted of 60 mice, which were allocated into five groups using a computer-generated randomization sequence, with 12 mice per group: the Sham group, I/R group, ESK + I/R group, ESK + chloroquine phosphate (CQ) + I/R group, and CQ + I/R group. The Sham group underwent the same anesthesia and procedural manipulations but without tourniquet application, whereas the other groups were all subjected to the left hindlimb I/R model. The doses of esketamine (10 mg/kg; Hengrui Pharmaceutical, Jiangsu, China) and chloroquine phosphate (60 mg/kg; Aladdin, Shanghai, China) were determined based on previous animal studies and the results of preliminary experiments [15,16]. These agents were administered intraperitoneally 20 min and 30 min before ischemia, respectively. The I/R and Sham groups received an equal volume of normal saline.

To accommodate the assessment of different outcome measures, the mice in each group were further divided into two subgroups (n = 6 each). Subgroup 1 was used for mechanical pain threshold testing, skeletal muscle contractile force measurement, and wet-to-dry weight ratio analysis. Subgroup 2 was used for the rotarod test, serum inflammatory cytokine assays, and histological and molecular biological analyses of the gastrocnemius muscle. On the day of model establishment, laser speckle blood perfusion imaging was performed in all groups before ischemia, after ischemia, and at 24 h after reperfusion. At 24 h after reperfusion, after completion of the mechanical pain threshold and skeletal muscle contractile force assessments, the left gastrocnemius muscle from subgroup 1 was harvested for wet-to-dry ratio analysis. After completion of the rotarod test, blood samples and the left gastrocnemius muscle were collected from subgroup 2 for the analysis of serum inflammatory cytokines, hematoxylin and eosin (H&E) staining, immunofluorescence, Western blotting, and reverse transcription-quantitative polymerase chain reaction (RT-qPCR). With the exception of blood collection, all tissue sampling procedures were performed after euthanasia.

An additional 10 mice were used as an independent animal cohort, with two mice per group. The same randomization procedure, treatment regimen, ischemia/reperfusion modeling protocol, and tissue collection time points were applied to increase the number of biological replicates for Western blot and RT-qPCR analyses.

### Establishment of the hindlimb I/R model

After induction of anesthesia with inhaled isoflurane, a McGivney hemorrhoidal ligator was used to apply an orthodontic rubber band to the proximal left hindlimb, thereby inducing ischemia of the left hindlimb for 3 h, followed by reperfusion for 24 h after release of the rubber band [17]. Throughout the ischemic period, anesthesia was maintained only during the application of the orthodontic rubber band, and body temperature was maintained at 37°C using a heating pad until the animals regained consciousness. Laser speckle imaging was performed after tourniquet application and before tourniquet release to confirm near-complete ischemia of the affected hindlimb, thereby verifying successful model establishment.

### Blood perfusion imaging

A laser speckle contrast imaging system was used to assess hindlimb blood perfusion in mice. After anesthesia with 1.5% inhaled isoflurane, the mice were placed on a black imaging platform, and blood perfusion in the plantar regions of both hind paws was measured using a blood perfusion imaging device (RFLSI III, RWD, Shenzhen, China). During the experiment, perfusion images were acquired before ischemic treatment, after tourniquet application, and at 24 h after tourniquet release and reperfusion. The images were analyzed using the accompanying software, and the level of blood perfusion was expressed as the perfusion ratio of the ischemic hindlimb to the contralateral healthy hindlimb.

### Rotarod test

Mice underwent rotarod pre-acclimation training for 3 consecutive days before model establishment, and performance was assessed using a mouse rotarod fatigue apparatus (ZS-RDM-XS, Zhongshi Dichuang, Beijing, China). The pre-acclimation training was performed in the accelerating mode, with an initial speed of 4 revolutions per minute (rpm), an acceleration of 20 rpm/min, and a maximum speed of 30 rpm for 5 min; each mouse was trained twice daily. Formal testing was conducted at 24 h after reperfusion, and the latency to fall was recorded using the same accelerating protocol. Each mouse was tested three times, and the mean value was used for statistical analysis. After the rotarod test, the mice were allowed to rest for 1 h before subsequent experiments.

### Mechanical pain threshold assessment

Baseline mechanical pain threshold measurements were performed for 3 consecutive days before model establishment. Prior to testing, mice were placed in transparent chambers with a metal mesh floor and allowed to acclimate for 1 h. The plantar surface of the affected hind paw was then stimulated perpendicularly using von Frey filaments (Touch Test, North Coast Medical, Gilroy, CA, USA), and the 50% paw withdrawal threshold was determined using the up-down method [18]. Formal testing was conducted again at 24 h after reperfusion using the same procedure.

### Skeletal muscle contractile force measurement

At 24 h after reperfusion, skeletal muscle contractile force was measured in mice. Under isoflurane anesthesia, the mice were placed in the prone position, and body temperature was maintained at 37°C using a heating pad. The left gastrocnemius muscle was kept continuously moistened with warm normal saline, and the distal tendon of the left gastrocnemius was connected to a high-precision force transducer (ADInstruments, Colorado Springs, CO, USA). After incision of the left biceps femoris to expose the sciatic nerve, the nerve was transected, and the distal nerve stump was stimulated using bipolar platinum electrodes to evoke gastrocnemius contraction. The parameters for nerve stimulation were as follows: twitch contraction, 10 V, 1 Hz, 1 ms; tetanic contraction, 10 V, 100 Hz, 1 ms, sustained for 2 s. For direct muscle stimulation, the electrodes were placed at the proximal end of the gastrocnemius, and the maximal force of twitch contraction (20 V, 1 Hz, 1 ms) and tetanic contraction (20 V, 100 Hz, 1 ms, sustained for 2 s) was recorded, with an interval of 2 min between each stimulation. Muscle contractile force data were recorded and analyzed using a PowerLab 8/30 data acquisition system and LabChart 8 software (ADInstruments, Colorado Springs, CO, USA).

### Skeletal muscle wet-to-dry weight ratio

At 24 h after reperfusion, the intact left gastrocnemius muscle was harvested from each mouse. After gently removing surface blood and moisture, the wet weight was measured. The tissue was then dried in an oven at 80°C for 48 h until a constant weight was reached, and the dry weight was recorded. The wet-to-dry weight ratio was subsequently calculated to assess the degree of tissue edema.

### Measurement of inflammatory cytokine levels

At 24 h after reperfusion, orbital blood was collected from the mice, and serum was isolated by centrifugation and stored at −80°C until further analysis. Serum levels of interleukin-1 beta (IL-1β), interleukin-6 (IL-6), and tumor necrosis factor-alpha (TNF-α) were measured using chemiluminescent immunoassay kits purchased from Wuhan Servicebio Technology Co., Ltd. (catalog numbers: GLM0010-100T, GLM0005-100T, and GLM0004-100T). All procedures were performed strictly in accordance with the manufacturers’ instructions.

### Assessment of skeletal muscle injury

At 24 h after reperfusion, the left gastrocnemius muscle was harvested from the mice for histological evaluation. The tissue was fixed in 4% paraformaldehyde, embedded in paraffin, and sectioned transversely. After deparaffinization and rehydration, the sections were subjected to H&E staining, and images were observed and acquired under a bright-field microscope.

### Western blot analysis

At 24 h after reperfusion, samples of the left gastrocnemius muscle were collected from the mice and stored at −80°C. The gastrocnemius tissue was homogenized and lysed in RIPA buffer, followed by centrifugation at 12,000 g for 20 min at 4°C to collect the total protein in the supernatant. Protein concentration was then determined using a BCA protein assay kit (Servicebio, Wuhan, China). One-fourth volume of 5 × loading buffer was added to the protein samples, which were then denatured at 95°C for 10 min. Equal amounts of total protein (20 μg/lane) were subjected to sodium dodecyl sulfate–polyacrylamide gel electrophoresis and subsequently transferred onto polyvinylidene difluoride membranes by constant-current electrophoretic transfer. After transfer, the membranes were blocked with a protein-free rapid blocking solution for 20 min. According to the molecular weight of the target proteins, the membranes were cut and incubated separately with the following primary antibodies overnight at 4°C: rabbit anti-microtubule-associated protein 1 light chain 3 II/I (LC3-II/I) antibody (HUABIO, Hangzhou, China; ET1701−65; 1:2000), rabbit anti-sequestosome 1 (p62) antibody (HUABIO, Hangzhou, China; HA721171; 1:2000), rabbit anti-mammalian target of rapamycin (mTOR) antibody (HUABIO, Hangzhou, China; ET1608−5; 1:5000), rabbit anti-phosphorylated mTOR (p-mTOR) antibody (HUABIO, Hangzhou, China; HA60094; 1:1000), and rabbit anti-β-actin antibody (Servicebio, Wuhan, China; GB15003; 1:5000). After washing with Tris-buffered saline containing Tween, the membranes were incubated for 2 h at room temperature with horseradish peroxidase-conjugated goat anti-rabbit immunoglobulin G (IgG) secondary antibody (Servicebio, Wuhan, China; GB23303; 1:10000). After further washing, protein bands were visualized using a chemiluminescent substrate (Applygen, Beijing, China; P1050), and band densitometric analysis was performed using ImageJ software, with β-actin as the internal reference for normalization.

### Immunofluorescence

At 24 h after reperfusion, the left gastrocnemius muscle was collected from the mice for immunofluorescence analysis. Gastrocnemius injury was evaluated by laminin immunostaining combined with detection of endogenous IgG extravasation [19]. Fresh gastrocnemius tissue was embedded in Optimal Cutting Temperature compound and stored at −80°C, and 10 μm transverse sections of the gastrocnemius were subsequently prepared.

For staining, the sections were washed with phosphate-buffered saline (PBS) and then blocked for 1 h at room temperature with 5% bovine serum albumin containing 0.3% Triton X-100. After removal of the blocking solution, the sections were incubated overnight at 4°C with a primary antibody against laminin (HUABIO, Hangzhou, China; HA721532; 1:1000). On the following day, the sections were rewarmed to room temperature and washed with PBS. They were then incubated for 2 h at room temperature in the dark with the following fluorescent secondary antibodies: Alexa Fluor 488-conjugated goat anti-rabbit IgG (HUABIO, Hangzhou, China; HA1121; 1:500) for detection of the laminin signal, and Alexa Fluor 594-conjugated goat anti-mouse IgG (HUABIO, Hangzhou, China; HA1126; 1:500) for detection of endogenous IgG extravasation in the tissue. After PBS washing, the sections were mounted with an anti-fade mounting medium containing DAPI, and images were observed and acquired under a fluorescence microscope.

All immunofluorescence images were acquired using identical parameters. Quantitative analysis was performed using ImageJ software after background subtraction. For each mouse, two transverse gastrocnemius sections were analyzed. From each image, 30 muscle fibers with clear boundaries and intact structure were randomly selected, and the mean fluorescence intensity of endogenous IgG within the muscle fibers was measured. The average value from the two images was taken as the result for that mouse.

### RT-qPCR

At 24 h after reperfusion, the left gastrocnemius muscle was collected from the mice for RT-qPCR analysis. Total RNA was extracted using the FastPure Complex Tissue/Cell Total RNA Isolation Kit (Vazyme, Nanjing, China; RC113). Genomic deoxyribonucleic acid (DNA) contamination was removed and cDNA was synthesized according to the manufacturer’s instructions using HiScript III RT SuperMix for qPCR (+gDNA wiper) (Vazyme, Nanjing, China; R323). Real-time quantitative PCR was then performed on a QuantStudio 6 Flex Real-Time PCR System (96-well, 0.2 mL block) using the SYBR Green method with ChamQ Universal SYBR qPCR Master Mix (Vazyme, Nanjing, China; Q711). The total reaction volume was 20 μL per well, and primer concentrations were prepared according to the manufacturer’s recommended conditions. The amplification protocol was as follows: 50°C for 2 min and 95°C for 30 s, followed by 40 cycles of 95°C for 10 s and 60°C for 30 seconds (s). After amplification, melt curve analysis was performed under the following conditions: 95°C for 15 s, 60°C for 1 min, and then a gradual increase to 95°C at 0.05°C/s with continuous fluorescence signal acquisition to verify amplification specificity. Gene expression levels were analyzed using the 2^^-ΔΔCt^ method [20], with β-actin as the internal reference gene and the Sham group as the calibrator for relative expression. The primer sequences used are listed in Table 1.

**Table 1:** Primer sequences used for RT-qPCR.

Genes	Forward (5′ to 3′)	Reverse (5′ to 3′)
Casp1	TGCCTGGTCTTGTGACTTGG	GTCACCCTATCAGCAGTGGG
Ccl2	TGACCCCAAGAAGGAATGGG	ACCTTAGGGCAGATGCAGTT
Il10	GGTGAGAAGCTGAAGACCCTC	GCCTTGTAGACACCTTGGTCTT
Il1b	GCCACCTTTTGACAGTGATGAG	GACAGCCCAGGTCAAAGGTT
Nlrp3	ATTACCCGCCCGAGAAAGG	TCGCAGCAAAGATCCACACAG
β-actin	GATCAGCAAGCAGGAGTACGA	GGGTGTAAAACGCAGCTCA

Abbreviations: Casp1, caspase-1; Ccl2, C-C motif chemokine ligand 2; Nlrp3, NOD-like receptor family pyrin domain-containing 3.

### Statistical analysis

Data are presented as the mean ± standard deviation (SD). Statistical analyses were performed using GraphPad Prism 8.0 (GraphPad Software, USA). Under the assumptions of normal distribution and homogeneity of variance, comparisons among multiple groups were conducted using one-way analysis of variance, followed by Tukey’s post hoc multiple-comparisons test. A value of *P* < 0.05 was considered statistically significant. The researchers were blinded to the group allocation during outcome assessment and data analysis.

## Results

### ESK attenuated hyperperfusion at 24 h after hindlimb I/R

Laser speckle imaging was used to dynamically assess blood perfusion in both hindlimbs of mice. The results showed that blood perfusion in the ischemic hindlimb was markedly reduced in all groups during tourniquet application, whereas perfusion recovered and developed into a hyperperfusion state at 24 h after reperfusion.

**Fig 1:**
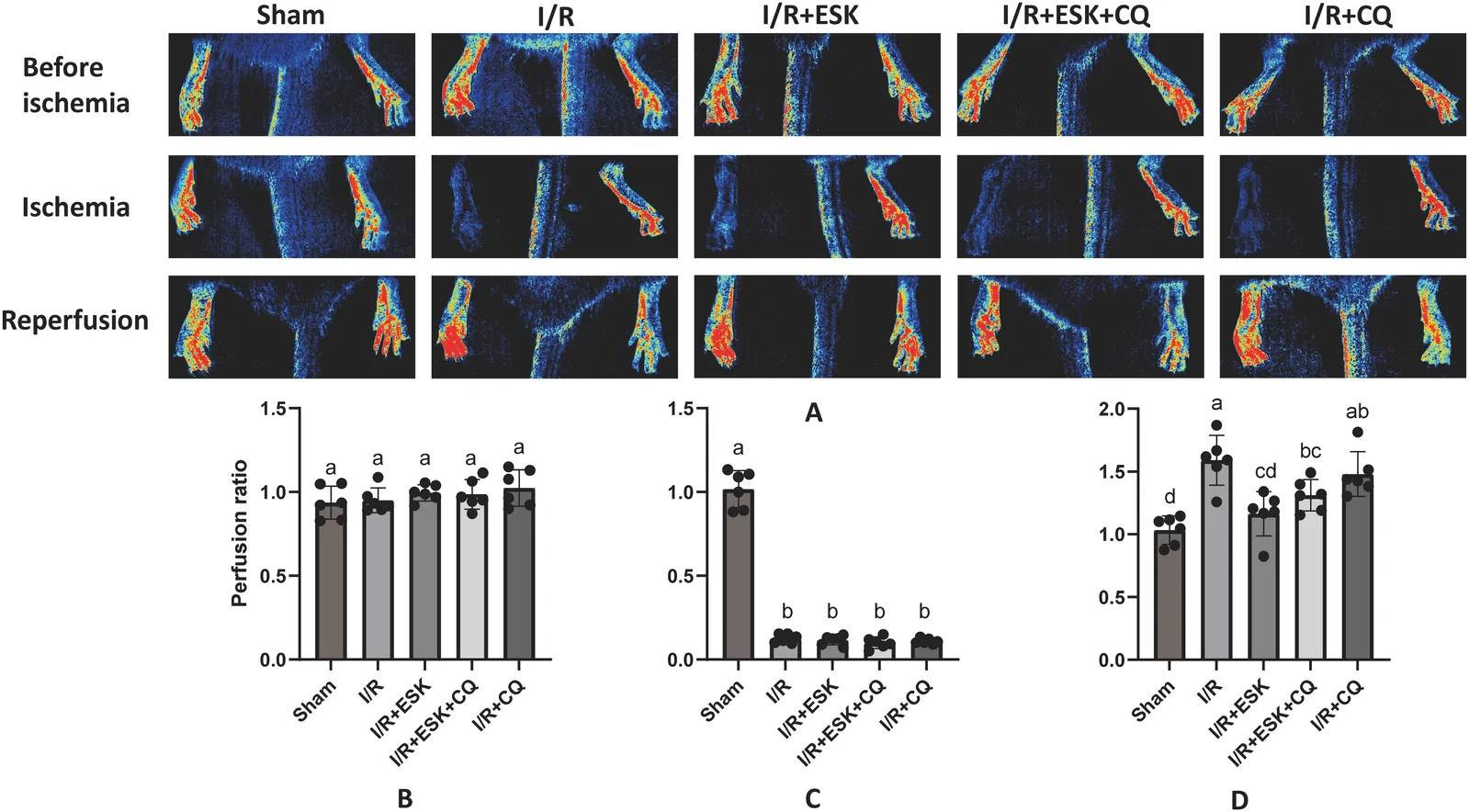
Effects of esketamine on blood perfusion after hindlimb ischemia-reperfusion in mice. (A) Laser speckle images at different stages, where red indicates high perfusion and blue indicates low perfusion.(B) Quantitative analysis before ischemia.(C) Quantitative analysis during ischemia.(D) Quantitative analysis at 24 h after reperfusion. Data are presented as the mean ± standard deviation (*n* = 6). Intergroup comparisons labeled with different letters indicate statistically significant differences (*P* < 0.05).

Before tourniquet application, there was no statistically significant difference in the hindlimb perfusion ratio (ischemic side/contralateral side) among the groups. During tourniquet application, the perfusion ratio of the ischemic hindlimb in each intervention group was significantly decreased compared with baseline, with no statistically significant difference among groups. At 24 h after reperfusion, the perfusion ratio in the I/R group was significantly higher than that in the Sham group, whereas ESK treatment significantly reduced this ratio. After co-administration of CQ, the effect of ESK showed a decreasing trend; CQ alone did not improve hyperperfusion. These findings suggest that ESK can attenuate the hyperperfusion state induced by I/R (Fig 1).

### ESK improved motor function at 24 h after I/R

The rotarod test showed that the latency to fall was significantly shorter in the I/R group than in the Sham group, whereas ESK treatment significantly prolonged the latency to fall. This improvement was abolished by co-administration of CQ, and CQ alone did not improve motor function. These findings indicate that ESK can improve motor function after I/R.

**Fig 2:**
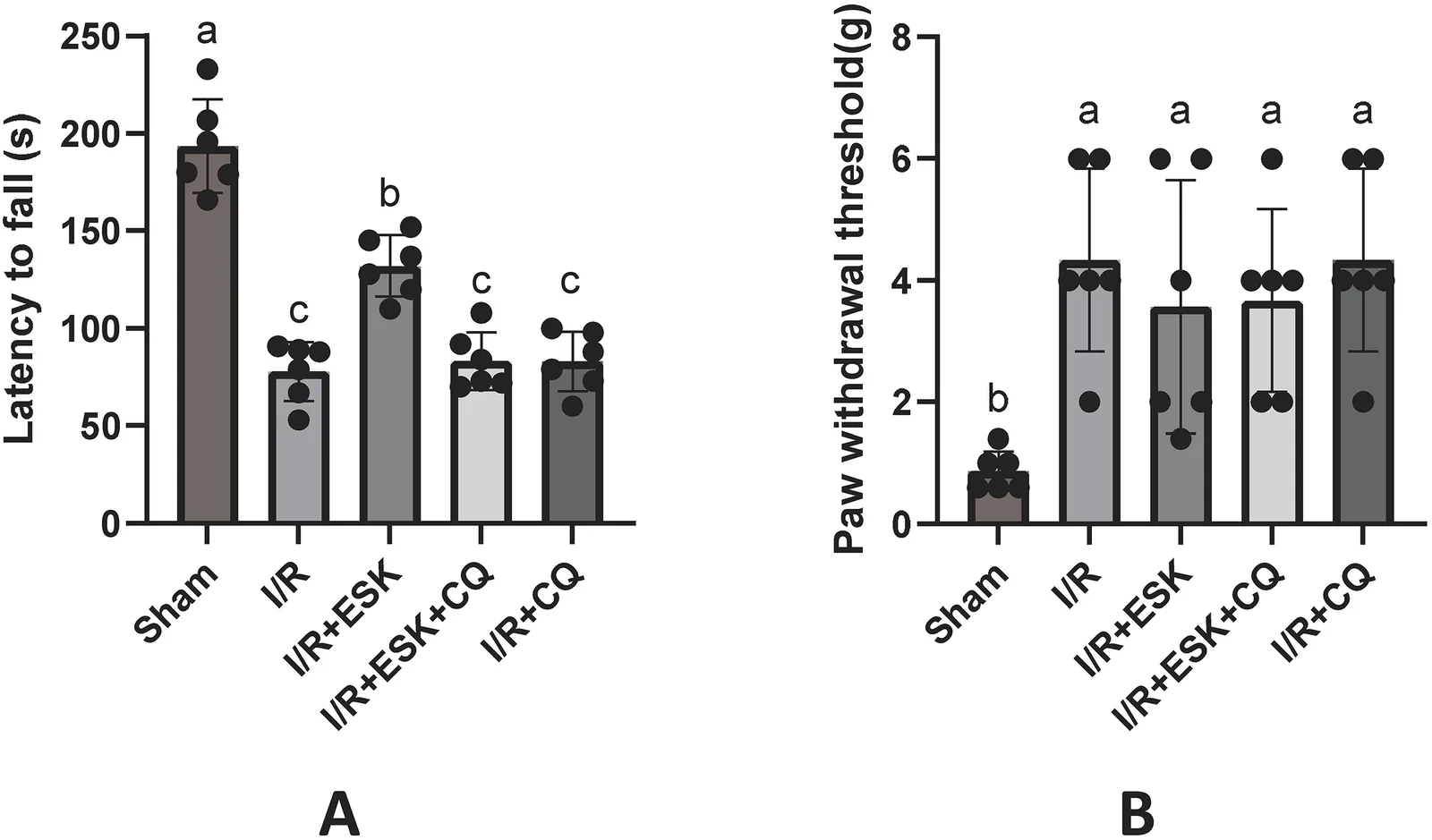
Effects of esketamine on motor performance and mechanical sensitivity after hindlimb ischemia-reperfusion in mice. (A) Rotarod test results, in which motor coordination was represented by the latency to fall from the accelerating rotarod.(B) Mechanical nociception test results, in which the paw withdrawal threshold of the hind paw was measured using von Frey filaments to assess mechanical sensitivity.Data are presented as the mean ± standard deviation (*n* = 6). Intergroup comparisons labeled with different letters indicate statistically significant differences (*P* < 0.05).

The von Frey test showed that the 50% paw withdrawal threshold in the Sham group was lower than that in all other groups, whereas no statistically significant differences were observed among the I/R-treated groups. These results indicate that the mechanical withdrawal threshold was elevated at 24 h after reperfusion, and that neither ESK nor CQ treatment significantly affected this parameter (Fig 2).

### ESK improved muscle contractile force at 24 h after I/R

Under direct muscle stimulation, both twitch force and tetanic force were significantly lower in the I/R group than in the Sham group, whereas ESK treatment partially restored both parameters. This improvement was attenuated after co-administration of CQ, while CQ alone produced no significant improvement. These findings indicate that ESK can partially ameliorate the decline in muscle contractile function induced by I/R, and that CQ can weaken this effect.

**Fig 3:**
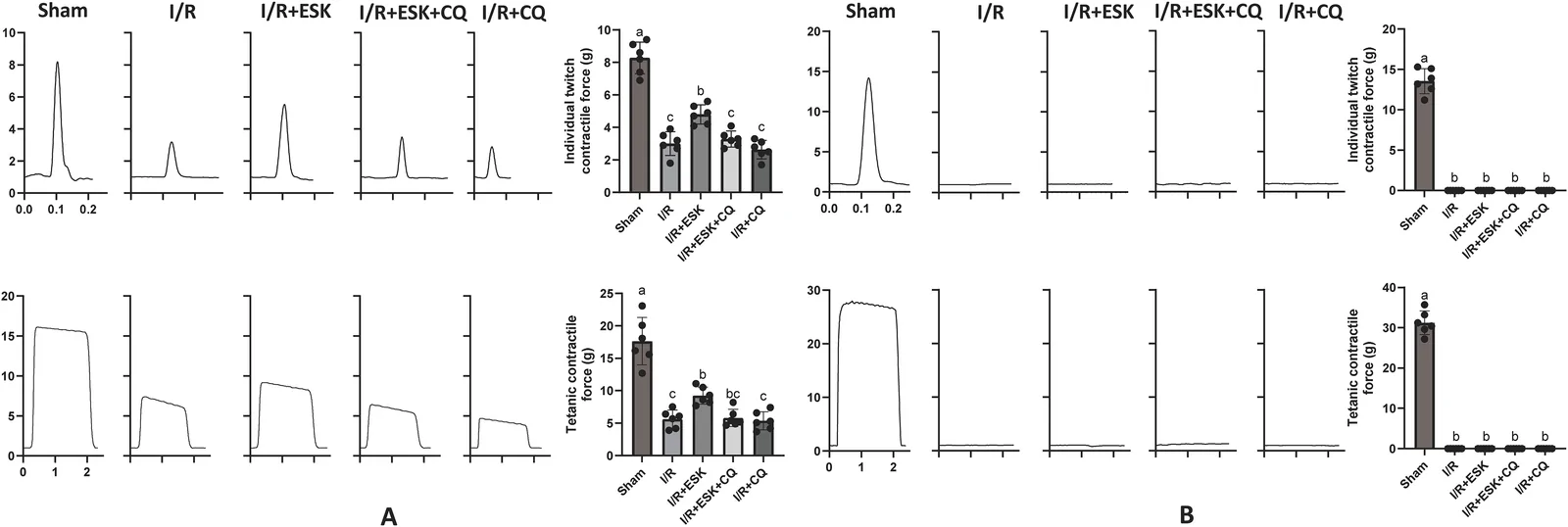
Effects of esketamine on muscle contractile force after hindlimb ischemia-reperfusion in mice. (A) Twitch contraction, tetanic contraction, and quantitative analysis under electrical stimulation of the gastrocnemius muscle.(B) Twitch contraction, tetanic contraction, and quantitative analysis under electrical stimulation of the sciatic nerve.Data are presented as the mean ± standard deviation (*n* = 6). Intergroup comparisons labeled with different letters indicate statistically significant differences (*P* < 0.05).

Under sciatic nerve electrical stimulation, the evoked twitch force and tetanic force were extremely low in all groups except the Sham group, with no statistically significant differences among groups. These results suggest that at 24 h after reperfusion, ESK did not restore nerve stimulation-driven muscle contraction, and CQ treatment did not alter this trend (Fig 3).

### ESK attenuated I/R-induced skeletal muscle histopathological injury, edema, and disruption of sarcolemmal integrity

H&E staining showed that, in the Sham group, gastrocnemius muscle fibers were regularly arranged, structurally intact, and separated by relatively small interstitial spaces. In contrast, the I/R group exhibited marked muscle injury, characterized by disorganized muscle fiber arrangement, widened interstitial spaces, and aggravated structural disruption. These histopathological changes were alleviated in the I/R + ESK group, as evidenced by relatively preserved muscle fiber morphology and reduced interstitial expansion. In the I/R + ESK + CQ group, however, structural damage and interstitial expansion were more pronounced than those in the I/R + ESK group, while the I/R + CQ group still displayed obvious histological injury.

To further quantify tissue edema, the wet-to-dry weight ratio of the gastrocnemius muscle was measured. The results showed that the wet-to-dry ratio was significantly increased in the I/R group compared with the Sham group, whereas ESK treatment significantly reduced this ratio. This beneficial effect was attenuated after co-administration of CQ, while the wet-to-dry ratio remained at a relatively high level in the CQ-alone group.

**Fig 4:**
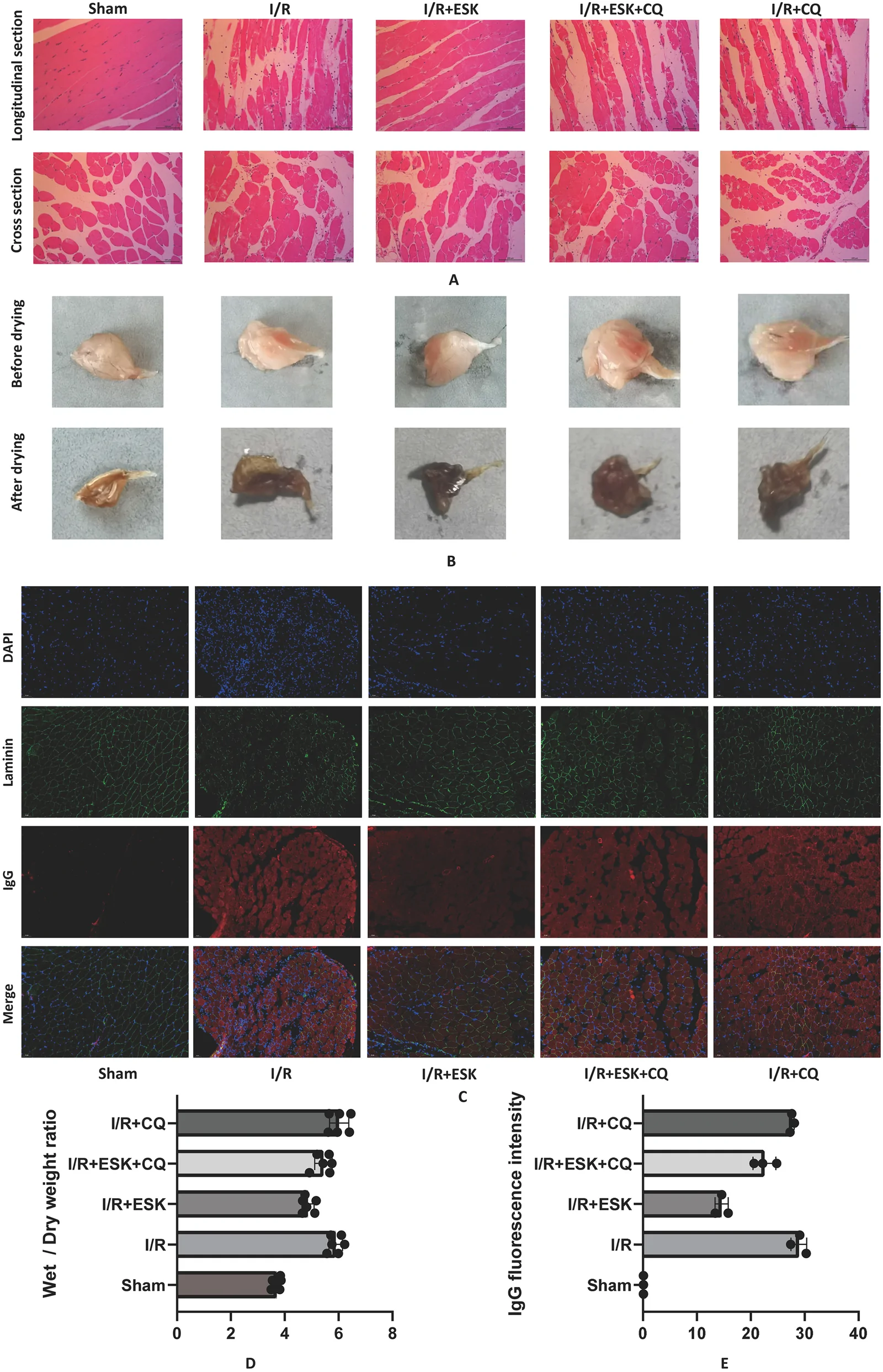
Effects of esketamine on skeletal muscle histopathology, edema, and sarcolemmal integrity after hindlimb ischemia-reperfusion. (A) Longitudinal and transverse H&E-stained sections of the gastrocnemius muscle.(B) Representative images of the gastrocnemius muscle from each group before and after drying.(C) Immunofluorescence images of transverse gastrocnemius sections showing DAPI, IgG, Laminin, and merged staining in each group.(D) Quantitative analysis of the wet-to-dry weight ratio of the gastrocnemius muscle (*n* = 6).(E) Quantitative analysis of IgG fluorescence intensity (*n* = 3).Data are presented as the mean ± standard deviation. Intergroup comparisons labeled with different letters indicate statistically significant differences (*P* < 0.05).

Laminin staining combined with endogenous IgG immunofluorescence was used to evaluate skeletal muscle membrane integrity. The results showed that almost no IgG-positive signal was observed within muscle fibers in the Sham group, whereas IgG fluorescence intensity was markedly increased in the I/R group. ESK treatment significantly reduced IgG extravasation, and this effect was partially attenuated by co-administration of CQ; CQ alone did not produce an obvious improvement. Taken together, these findings indicate that ESK can attenuate I/R-induced gastrocnemius tissue injury, edema, and disruption of sarcolemmal integrity, whereas CQ can partially weaken its protective effects (Fig 4).

### ESK attenuated the systemic inflammatory response induced by I/R and downregulated the transcription of certain inflammation-related genes

To evaluate the systemic inflammatory response induced by I/R, serum levels of TNF-α, IL-6, and IL-1β were measured in each group using chemiluminescent immunoassay. The results showed that serum TNF-α and IL-6 levels were significantly elevated in the I/R group, whereas ESK treatment significantly reduced both. This anti-inflammatory effect was attenuated after co-administration of CQ. In contrast, no significant differences in IL-1β levels were observed among the groups.

**Fig 5:**
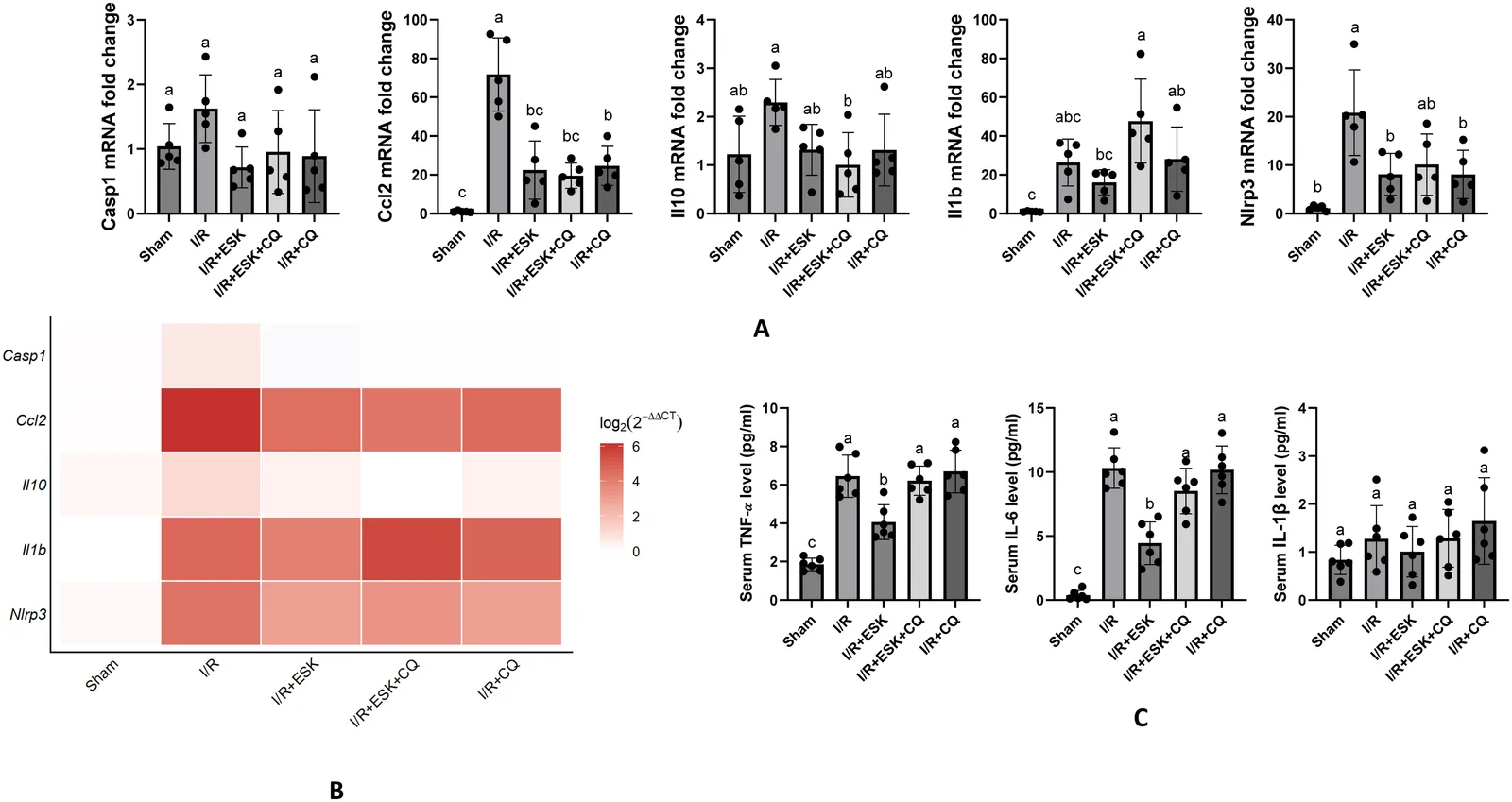
Effects of esketamine on the systemic inflammatory response and inflammation-related gene expression after hindlimb ischemia-reperfusion. (A) mRNA expression levels of target genes in each group, normalized to the internal reference gene and presented as relative expression levels (*n* = 5). (B) Heatmap of the relative expression levels of target genes in each group. Data are shown as log_2_(2^^-ΔΔCT^), and color variation indicates the relative level of gene expression. (C) Serum levels of TNF-α, IL-6, and IL-1β in each group are also shown (*n* = 6).Data are presented as the mean ± standard deviation. Intergroup comparisons labeled with different letters indicate statistically significant differences (*P* < 0.05).

RT-qPCR was further performed to assess the transcript levels of inflammation-related genes in the gastrocnemius muscle. The results showed no statistically significant difference in *Casp1* mRNA expression among the groups. Compared with the Sham group, the I/R group exhibited increased mRNA expression of *Ccl2* and *Nlrp3*. Following ESK treatment, the expression levels of these genes were reduced relative to those in the I/R group. After combined treatment with CQ, *Il1b* mRNA expression was significantly increased, whereas *Ccl2, Il10*, and *Nlrp3* mRNA expression showed no consistent trend (Fig 5).

### ESK suppressed mTOR phosphorylation and induced autophagy-related changes, which were attenuated by CQ

To clarify the effect of ESK on autophagy-related pathways after I/R, the expression of autophagy-related proteins in the gastrocnemius muscle was assessed at 24 h of reperfusion. The results showed that, compared with the I/R group, ESK treatment increased the LC3 II/I ratio, decreased p62 expression, and reduced the p-mTOR/mTOR ratio, suggesting that ESK treatment is associated with inhibition of mTOR signaling and alterations in the expression of autophagy-related proteins.

**Fig 6:**
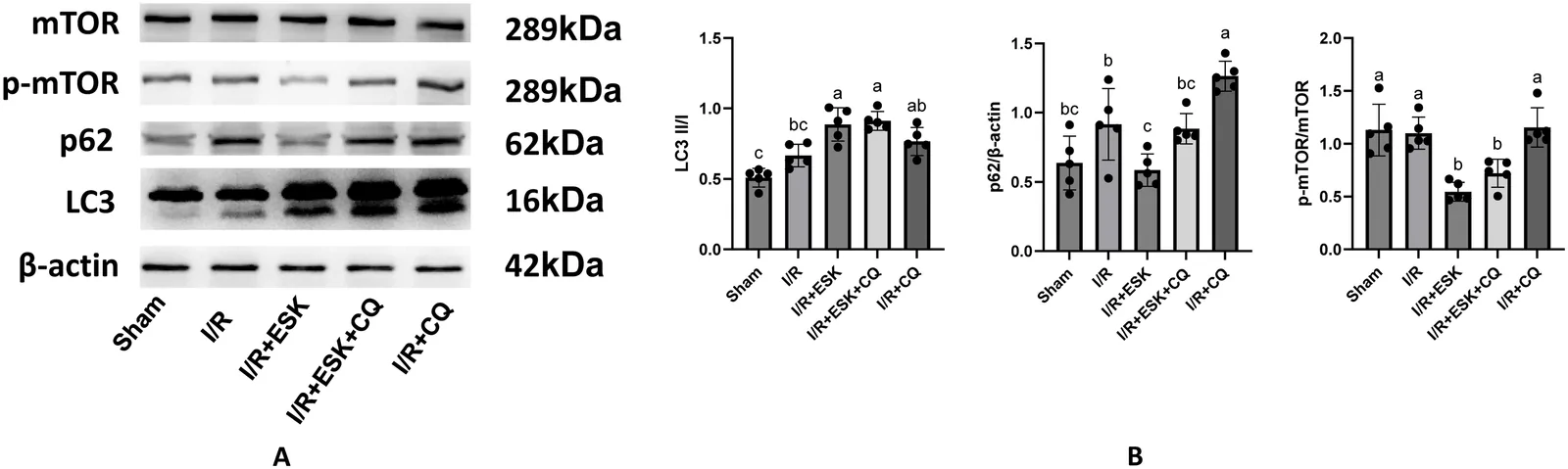
Effects of esketamine on autophagy-related proteins in skeletal muscle after hindlimb ischemia-reperfusion in mice. (A) Representative immunoblot bands of each protein.(B) Densitometric quantification of each protein.Data are presented as the mean ± standard deviation (*n* = 5). Intergroup comparisons labeled with different letters indicate statistically significant differences (*P* < 0.05).

Following co-treatment with CQ, the LC3-II/I ratio remained elevated, while p62 expression tended to increase compared with that in the ESK-alone group. The CQ-alone group similarly exhibited an elevated LC3-II/I ratio and marked p62 accumulation. These findings suggest that CQ may partially inhibit the ESK-associated autophagic degradation process, resulting in the accumulation of LC3-II and the autophagic substrate p62. (Fig 6).

## Discussion

The present study demonstrated that, during the acute phase of tourniquet-related hindlimb I/R injury, ESK attenuated tissue damage and improved certain functional outcomes. Its protective effects may be associated with the suppression of inflammation and the modulation of autophagy-related pathways.

The tourniquet-induced hindlimb I/R model has good clinical relevance. In orthopedic surgery, although tourniquets can reduce intraoperative blood loss and improve surgical field exposure, their release is often followed by local edema, amplified inflammation, and neuromuscular dysfunction, thereby affecting early postoperative mobilization and rapid recovery [4,21]. Particularly in the context of ERAS, 24 h after reperfusion (postoperative day 1) is often used as a clinical observation time point for evaluating tourniquet-related early recovery, local tissue changes, and functional outcomes [22,23]. Therefore, this study selected this time point for observation, which can, to some extent, reflect perioperative early tissue injury and the effects of intervention, and thus has certain translational significance.

In the present study, hyperperfusion was observed in the ischemic hindlimb at 24 h after I/R. This phenomenon more likely reflects microcirculatory imbalance in the setting of post-reperfusion inflammatory vasodilation, reactive hyperemia, and tissue edema, rather than a true improvement in perfusion recovery. Previous studies have suggested that, under conditions of active inflammation and marked tissue edema, local blood flow distribution, vascular permeability, and tissue optical properties may all be altered [24,25], thereby affecting laser speckle imaging readouts. ESK reduced this abnormal hyperperfusion, and, together with its effects in alleviating edema and inflammation, this finding suggests that ESK may help improve local microcirculatory disturbances after reperfusion.

Notably, the effects of ESK on different components of the neuromuscular system were not entirely uniform. ESK improved rotarod performance and partially restored contractile capacity under direct stimulation of the gastrocnemius muscle, but had limited effects on the mechanical withdrawal threshold and sciatic nerve stimulation-evoked muscle contraction. This suggests that, within the acute 24 h reperfusion window, neural dysfunction may still be prominent and may recover more slowly than local muscle function. In terms of motor output, the lack of clear recovery in nerve stimulation-induced contraction indicates that nerve-driven output remained restricted. On the sensory input side, the elevated mechanical withdrawal threshold suggests that the von Frey findings may not reflect typical mechanical hypersensitivity, but rather a reduction in sensation or a “numbness-like” change. These findings indicate that the protective effects of ESK during the acute phase may be more pronounced at the muscular level, while being insufficient to reverse neural dysfunction within a short period. Given that peripheral nerve injury often involves more complex processes, including impaired neural microcirculation, axonal and myelin damage, and compression caused by local edema, recovery generally depends more on time and structural reconstruction [26,27], which may explain this phenomenon.

The attenuation of skeletal muscle injury and tissue edema may constitute an important basis for the beneficial effects of ESK on early functional outcomes. Local edema developing after reperfusion not only disrupts the mechanical environment surrounding muscle fibers and limits contractile efficiency, but may also aggravate secondary injury by compressing the microcirculation and adjacent neural structures [28]. At the same time, IgG extravasation reflects not merely simple interstitial fluid retention, but also suggests impairment of sarcolemmal barrier integrity [29]. Therefore, the protective effects of ESK may involve not only the alleviation of inflammatory edema, but also the maintenance of sarcolemmal barrier stability and the restriction of abnormal plasma component entry into muscle fibers. The partial attenuation of this effect after co-administration of CQ further suggests that such structural protection may be related to autophagy-associated regulation.

Inflammation may represent an important entry point underlying the protective effects described above. After I/R, systemic inflammatory cytokines were elevated, accompanied by upregulated local transcription of inflammation-related genes, indicating that inflammatory responses play a key role in this model. ESK reduced the levels of certain inflammatory cytokines and the expression of inflammation-related genes, suggesting that it may suppress the sustained amplification of inflammation after reperfusion. Given that chemokine recruitment, inflammasome activation, and cytokine release can collectively lead to immune cell infiltration, endothelial activation, and microcirculatory dysfunction, this anti-inflammatory effect is likely closely associated with its ability to attenuate edema, improve the local tissue environment, and promote functional recovery [30,31]. It should be noted that CQ intervention produced inconsistent changes in the expression of different inflammation-related genes, suggesting a complex regulatory mechanism. In addition to being a classical autophagy inhibitor, CQ also exerts immunomodulatory and anti-inflammatory effects [32]. Therefore, the observed alterations in inflammation-related gene expression may have been jointly influenced by multiple confounding factors, including autophagy inhibition, the intrinsic pharmacological effects of CQ, and other related factors. Accordingly, the current findings only support the conclusion that ESK alleviates the inflammatory response, whereas the specific relationship between its anti-inflammatory effects and autophagy regulation remains difficult to delineate and requires further investigation.

The Western blot results suggest that the protective effects of ESK observed in this study may be associated with the inhibition of mTOR activation and the restoration of autophagy-related homeostasis in skeletal muscle. After I/R, if damaged mitochondria, abnormal proteins, and other injury-related intracellular components in skeletal muscle cells are not cleared in a timely manner, they may continuously trigger oxidative stress and amplify inflammation, thereby further disrupting local tissue structure and microenvironmental homeostasis [33,34]. At the same time, intensified inflammatory responses and cellular structural damage may increase vascular and sarcolemmal permeability, leading to tissue edema, loss of membrane integrity, and impaired functional recovery [7,35]. Therefore, autophagy in this process may represent not merely a simple intracellular degradation pathway, but also an important mechanism linking injury clearance, inflammation control, and structural protection [36]. Notably, mitochondrial dysfunction is also a key pathological feature of skeletal muscle I/R injury and may manifest as impaired mitochondrial respiration, reduced calcium retention capacity, and the accumulation of lipid peroxidation products [37]. Mitophagy, an important form of selective autophagy, plays a critical role in removing damaged mitochondria and maintaining mitochondrial quality control and organelle homeostasis [38]. Based on the present findings, ESK may protect against skeletal muscle I/R injury by regulating overall autophagy-related homeostasis. However, whether mitophagy and the regulation of mitochondrial homeostasis are involved in this protective effect remains to be further verified. Taken together, based on this mechanism, the findings of the present study may not be independent of one another, but rather reflect an integrated protective effect of ESK through improving autophagy-related processes and reducing secondary tissue injury. This suggests that the protective effect of ESK on skeletal muscle after I/R may not act separately on individual aspects such as inflammation, edema, or functional impairment; instead, it may more likely alleviate secondary inflammatory responses and tissue injury by restoring autophagy-related homeostasis, ultimately manifesting as reduced edema and improved muscle function. The partial attenuation of ESK’s protective effects after co-administration of CQ further indicates that the intervention of ESK in I/R injury may result from the combined action of multiple mechanisms, among which autophagy-related regulation may constitute an important component and may be closely associated with its anti-inflammatory effects and improvement of the local tissue microenvironment.

This study has several limitations. First, it focused primarily on an acute observation window of 24 h after reperfusion, which captured early inflammatory responses, edema, tissue injury, and functional impairment but did not determine whether the protective effects of ESK were sustained. Future studies should include longer-term observation time points to further evaluate its effects on skeletal muscle regeneration, fibrosis, and neuromuscular functional recovery. Second, this study mainly investigated the effects of ESK on inflammation and overall autophagy-related homeostasis in skeletal muscle, without directly assessing mitochondrial function or mitophagy-related markers. Therefore, whether ESK protects against I/R injury by regulating mitochondrial homeostasis requires further investigation. Third, although pharmacological intervention with CQ provided evidence supporting the involvement of autophagy, its multitarget effects limited further elucidation of the specific mechanisms. Finally, only a single dose and administration regimen were evaluated; thus, the optimal dose, timing of administration, and approach for integrating ESK into perioperative anesthesia and analgesia protocols remain to be determined.

In summary, this study demonstrates that, during the acute phase of tourniquet-related hindlimb I/R injury, ESK can attenuate early secondary skeletal muscle injury and improve certain functional outcomes. Its protective effects may be associated with the suppression of inflammation and the regulation of mTOR-related autophagy. These findings suggest that, beyond its role in perioperative analgesia and sedation, ESK may also possess a certain degree of tissue-protective potential and may provide a new perspective for the intervention of tourniquet-related I/R injury.

## Supporting information

S1 Data Blood perfusion raw data. (XLSX)

S2 Data Wet-to-dry weight ratio raw data. (XLSX)

S3 Data Rotarod test and mechanical pain threshold raw data. (XLSX)

S4 Data Muscle contractile force raw data. (XLSX)

S5 Data Inflammatory cytokine levels raw data. (XLSX)

S6 Data Western blot raw data. (XLSX)

S7 Data PCR raw data. (XLSX)

S8 Data Immunofluorescence raw data. (XLSX)

S1 Fig Uncropped and unadjusted Western blot images. (PDF)

S1 File The ARRIVE guidelines 2.0: author checklist. (PDF)
