# Supplementary material for: Esketamine exerts a protective effect against skeletal muscle injury induced by hindlimb ischemia-reperfusion in mice by regulating autophagy
Source: PLoS One. 2026 Sep 15;21(9):e0358125. doi: 10.1371/journal.pone.0358125 (PMC13577385; doi:10.1371/journal.pone.0358125)
Supplement: Supplementary file 1 [file pone.0358125.s009.pdf]

Replicate 1

The western blot images were obtained using an integrated chemiluminescence imaging system (ChemiScope S6, Clinx Scientific Instruments Co., Ltd., Shanghai, China). Asterisks (*) indicate the western blot images used for Fig. 6.

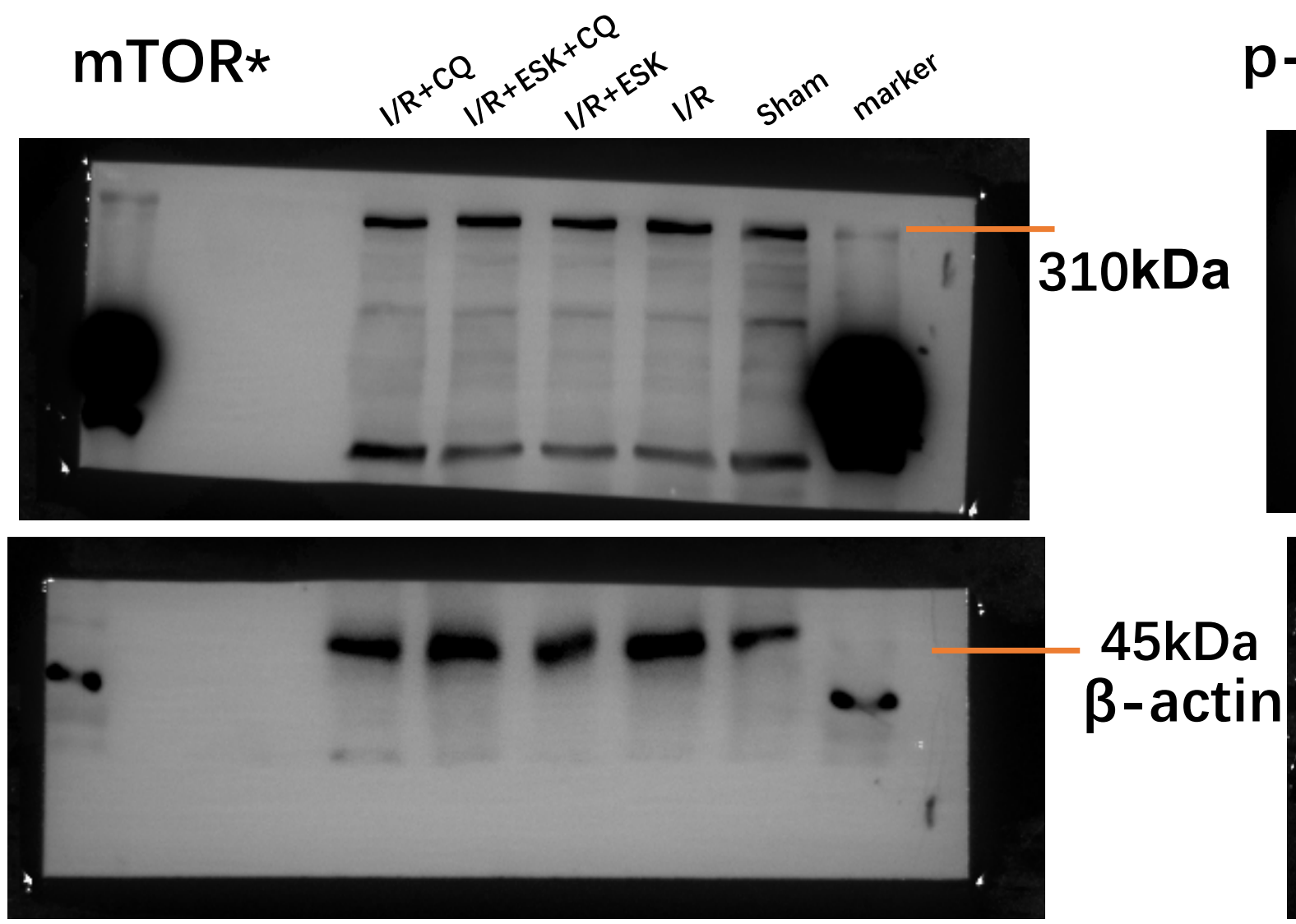

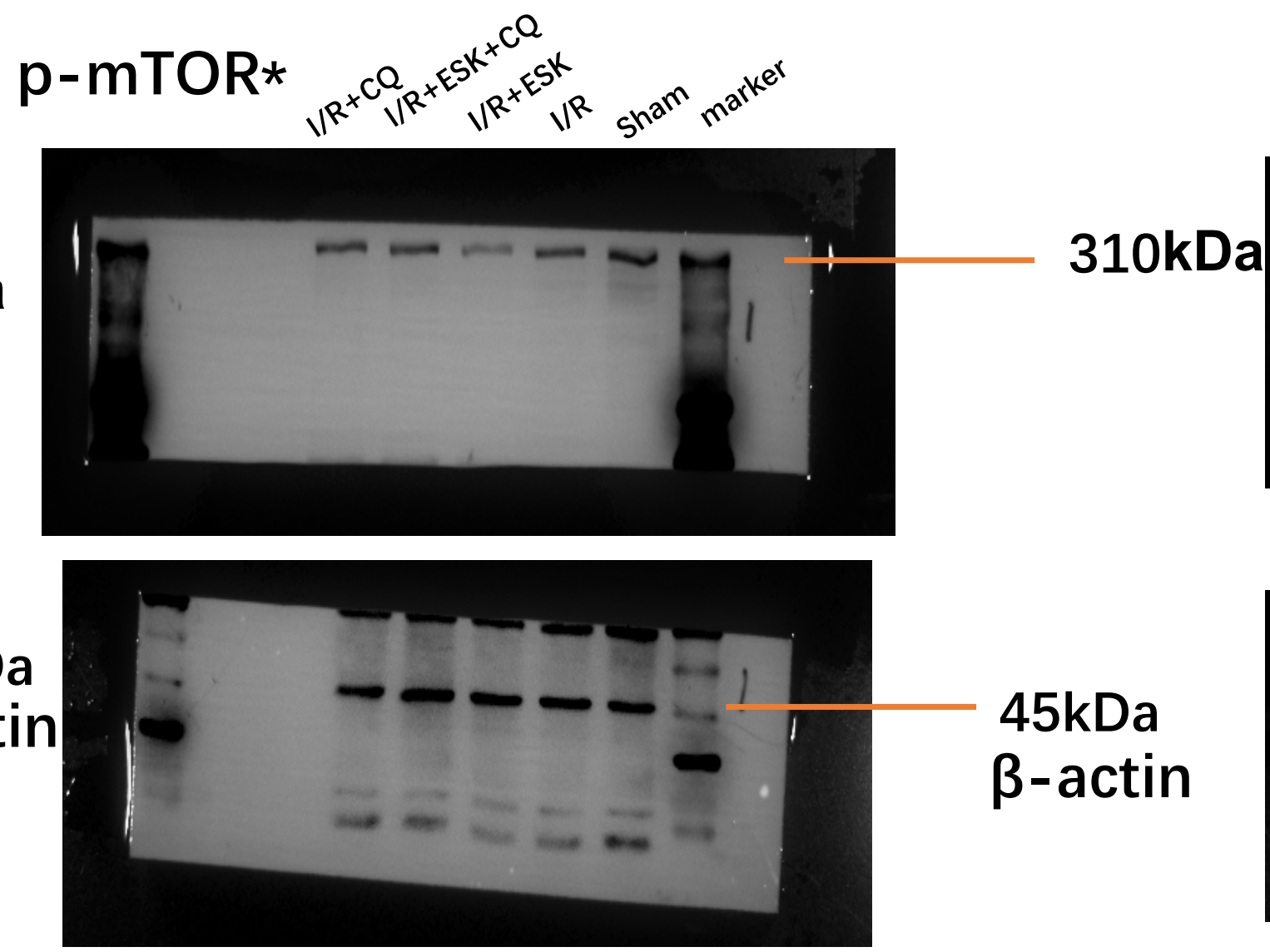

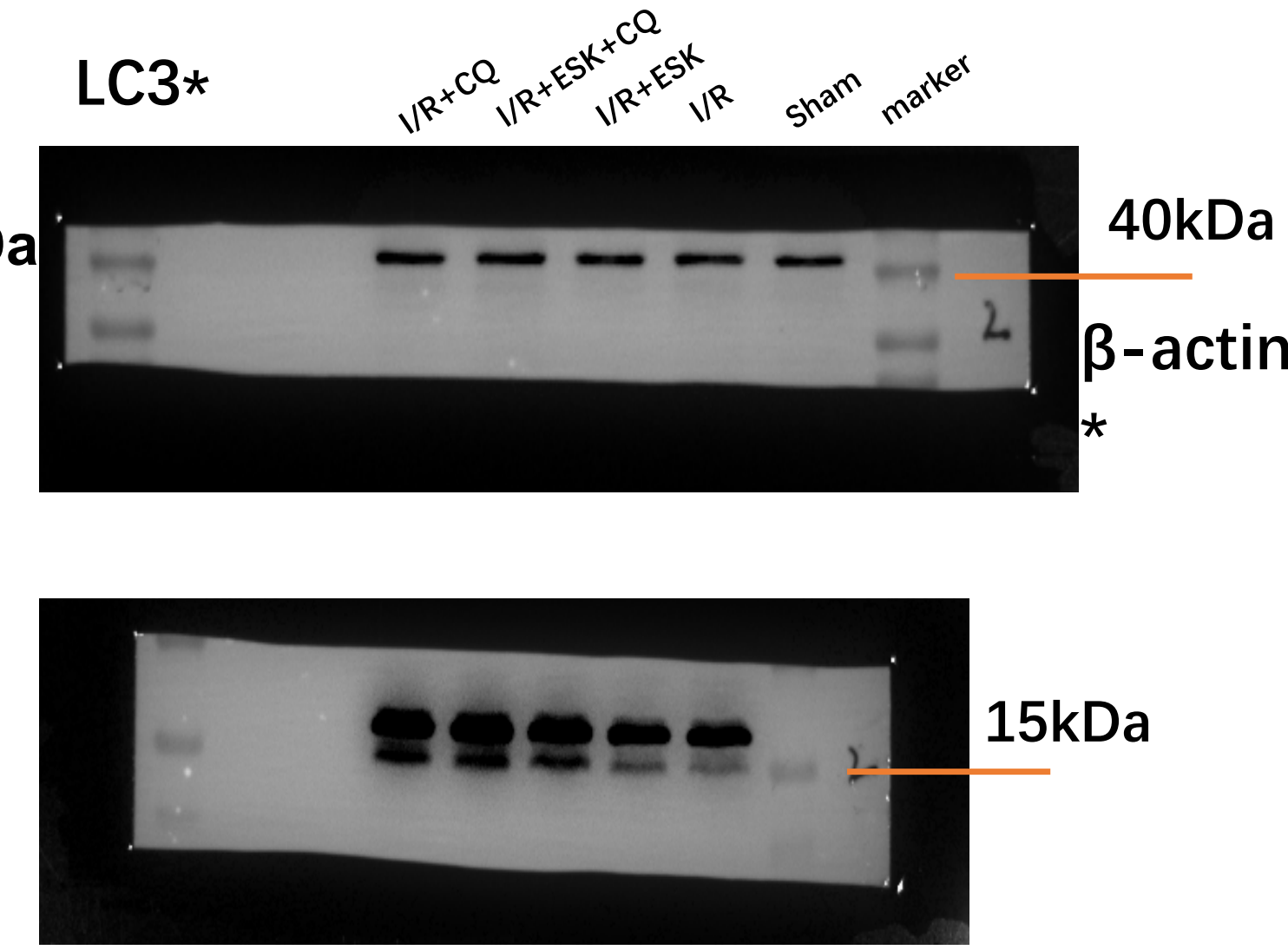

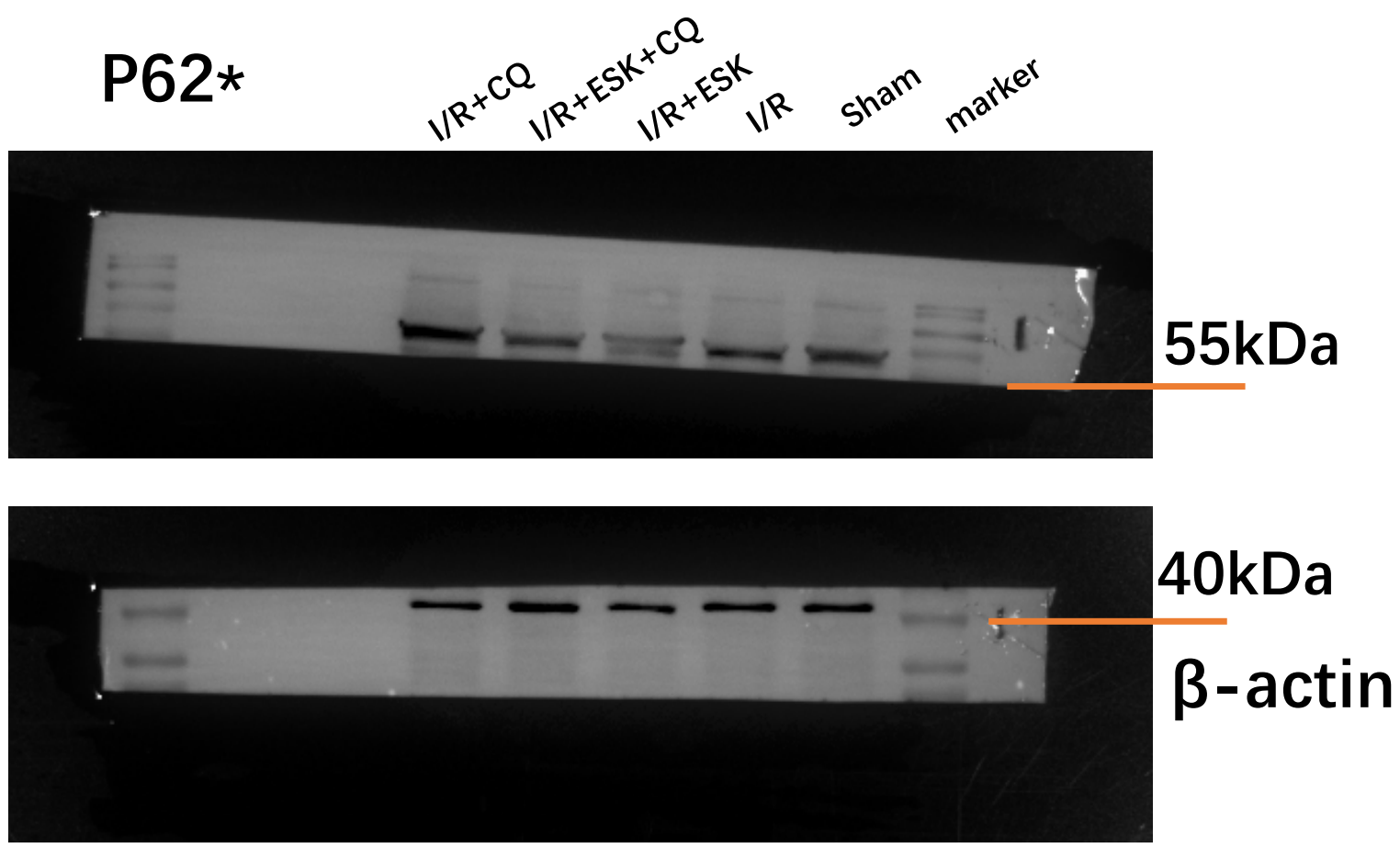

Replicate 2

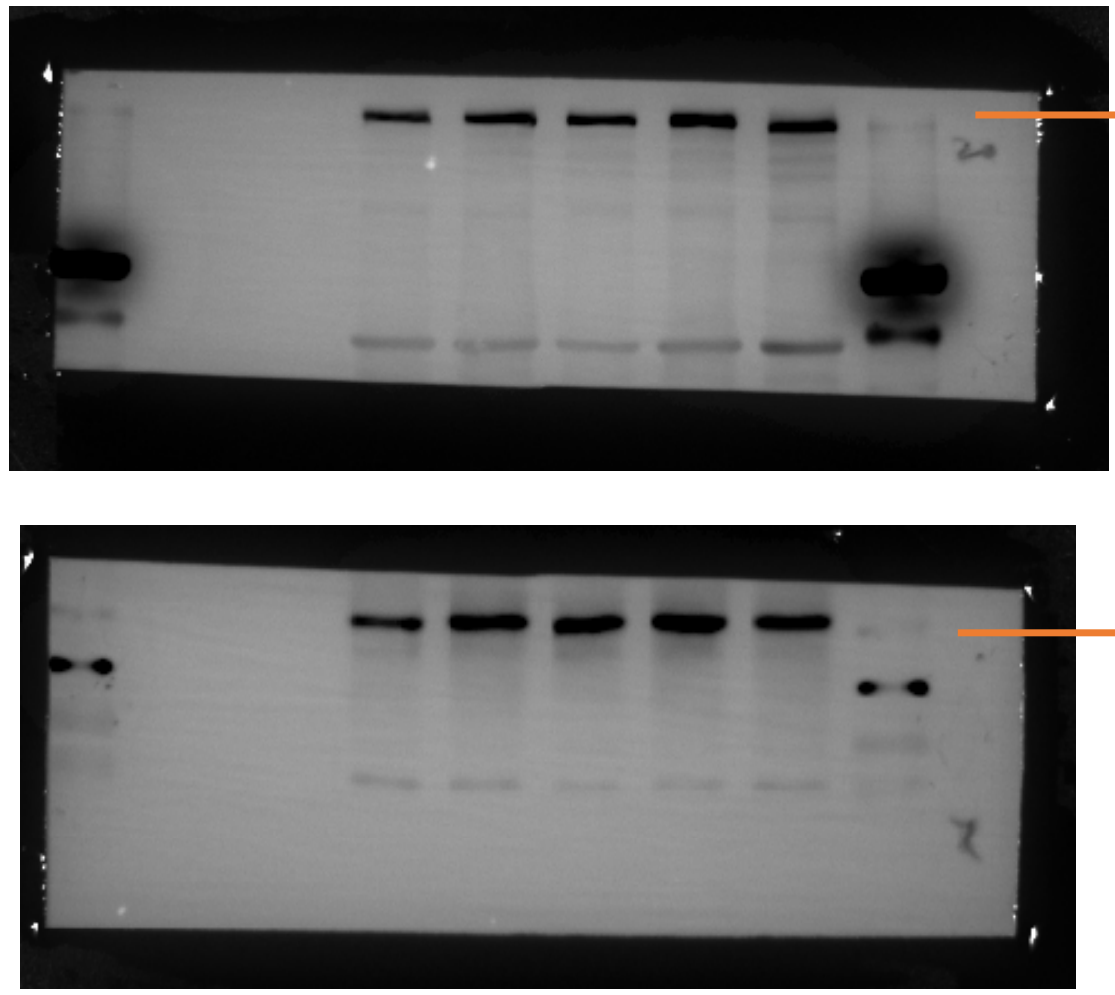

mTOR
I/R+CQ
I/R+ESK+CQ
I/R+ESK
I/R
Sham
marker
310kDa
45kDa
β-actin

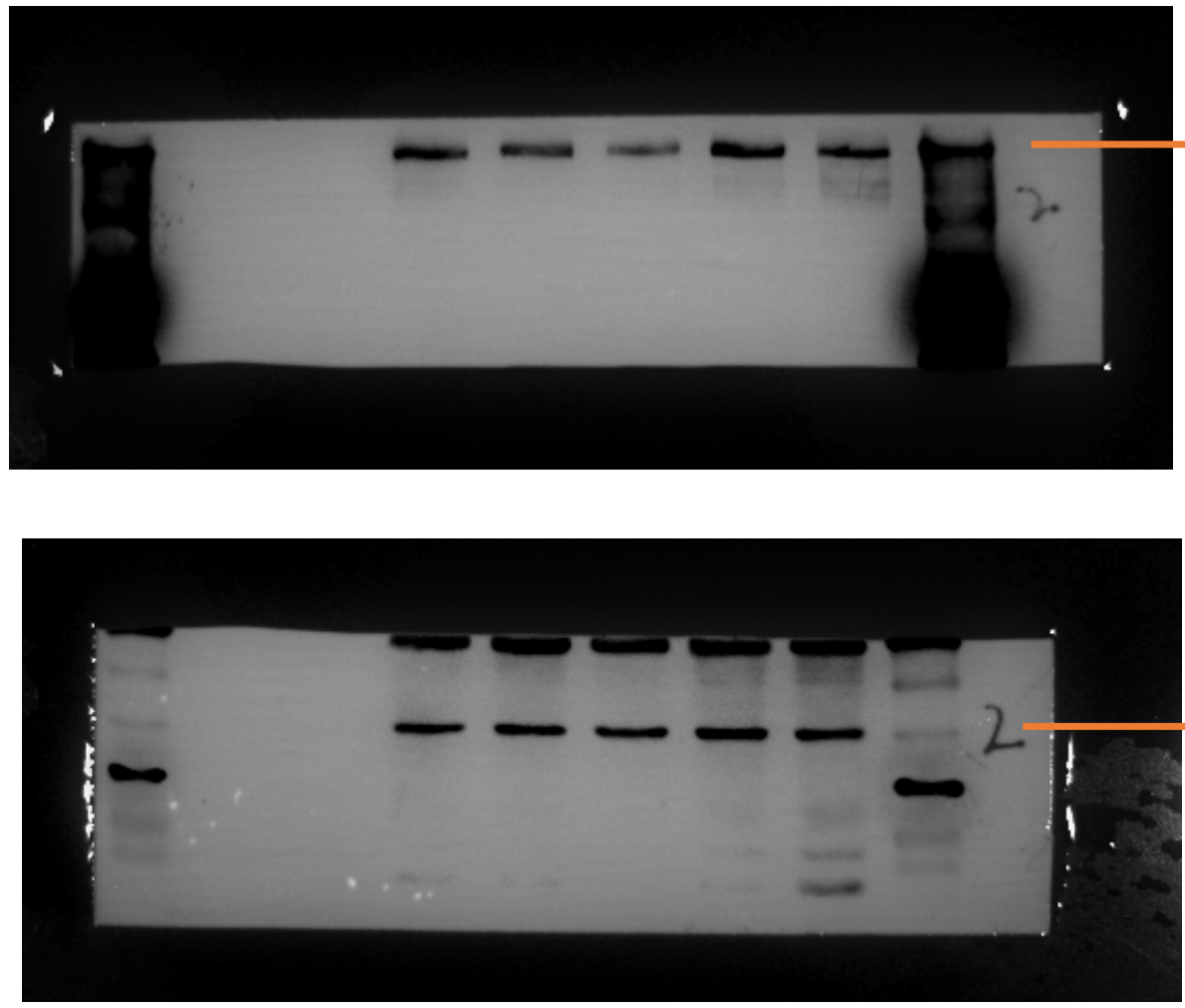

p-mTOR
I/R+CQ
I/R+ESK+CQ
I/R+ESK
I/R
Sham
marker
310kDa
45kDa
β-actin

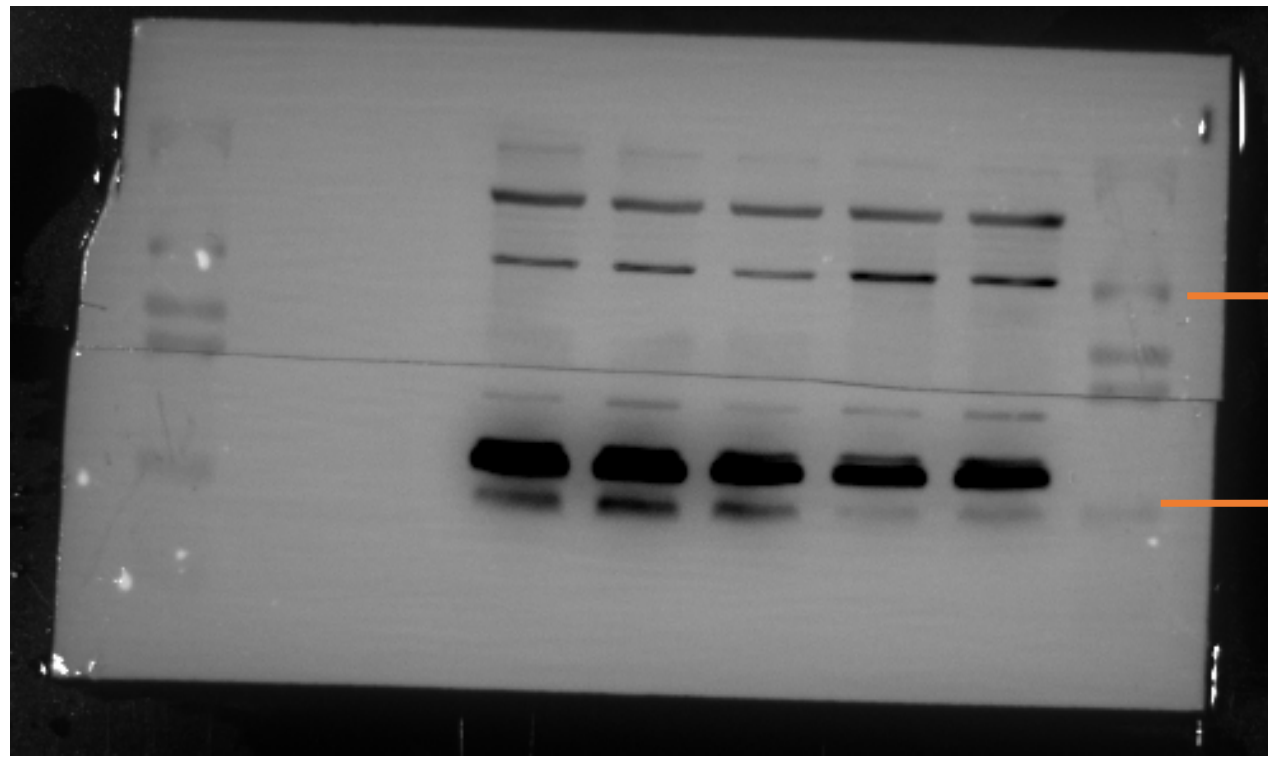

LC3
I/R+CQ
I/R+ESK+CQ
I/R+ESK
I/R
Sham
marker
β-actin
40kDa
15kDa

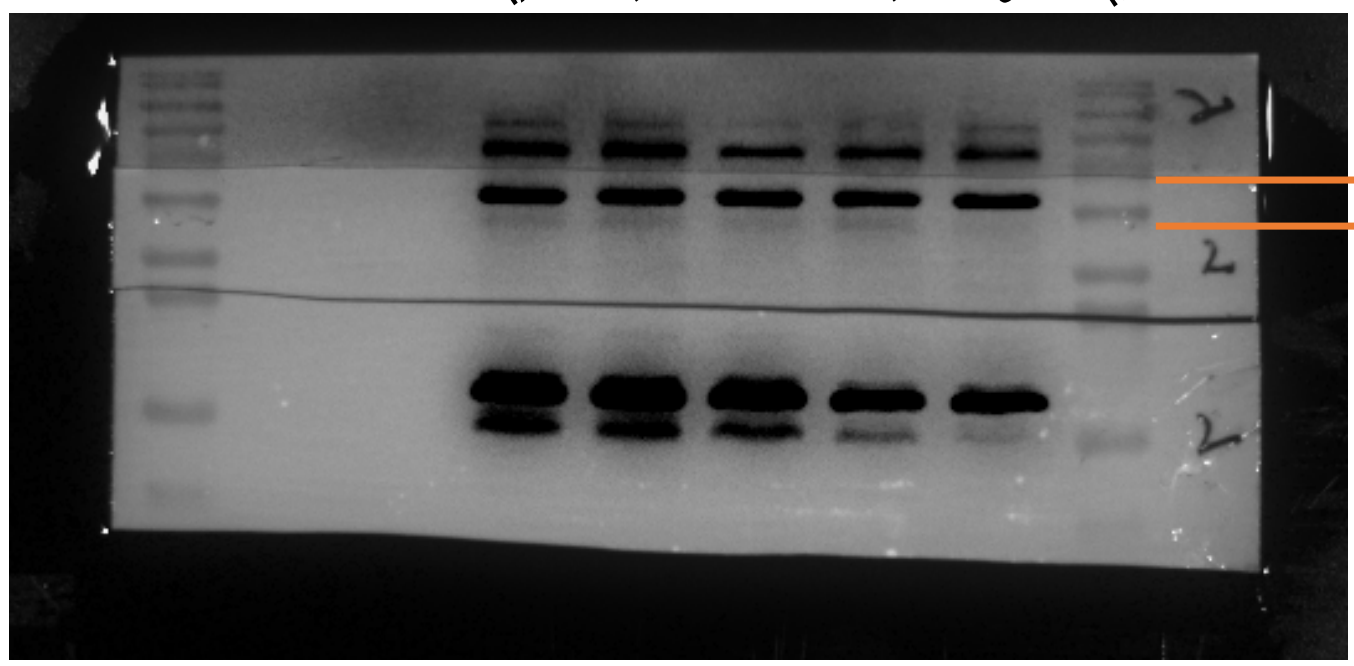

p62
I/R+CQ
I/R+ESK+CQ
I/R+ESK
I/R
Sham
marker
55kDa
β-actin
40kDa

Replicate 3

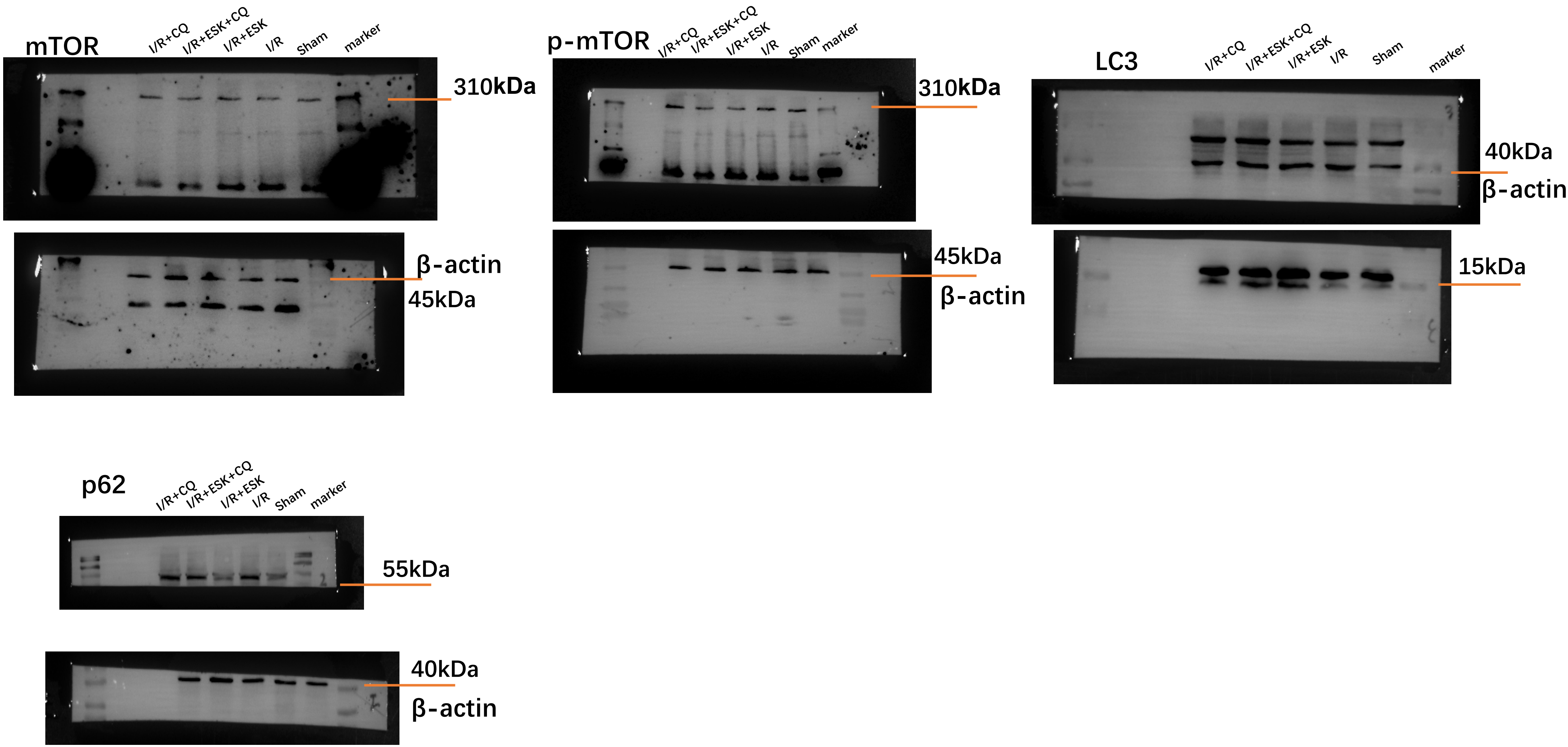
mTOR
I/R+CQ
I/R+ESK+CQ
I/R+ESK
I/R
Sham
marker
310kDa
β-actin
45kDa
p-mTOR
I/R+CQ
I/R+ESK+CQ
I/R+ESK
I/R
Sham
marker
310kDa
45kDa
β-actin
LC3
I/R+CQ
I/R+ESK+CQ
I/R+ESK
I/R
Sham
marker
40kDa
β-actin
15kDa
p62
I/R+CQ
I/R+ESK+CQ
I/R+ESK
I/R
Sham
marker
55kDa
40kDa
β-actin

Replicate 4

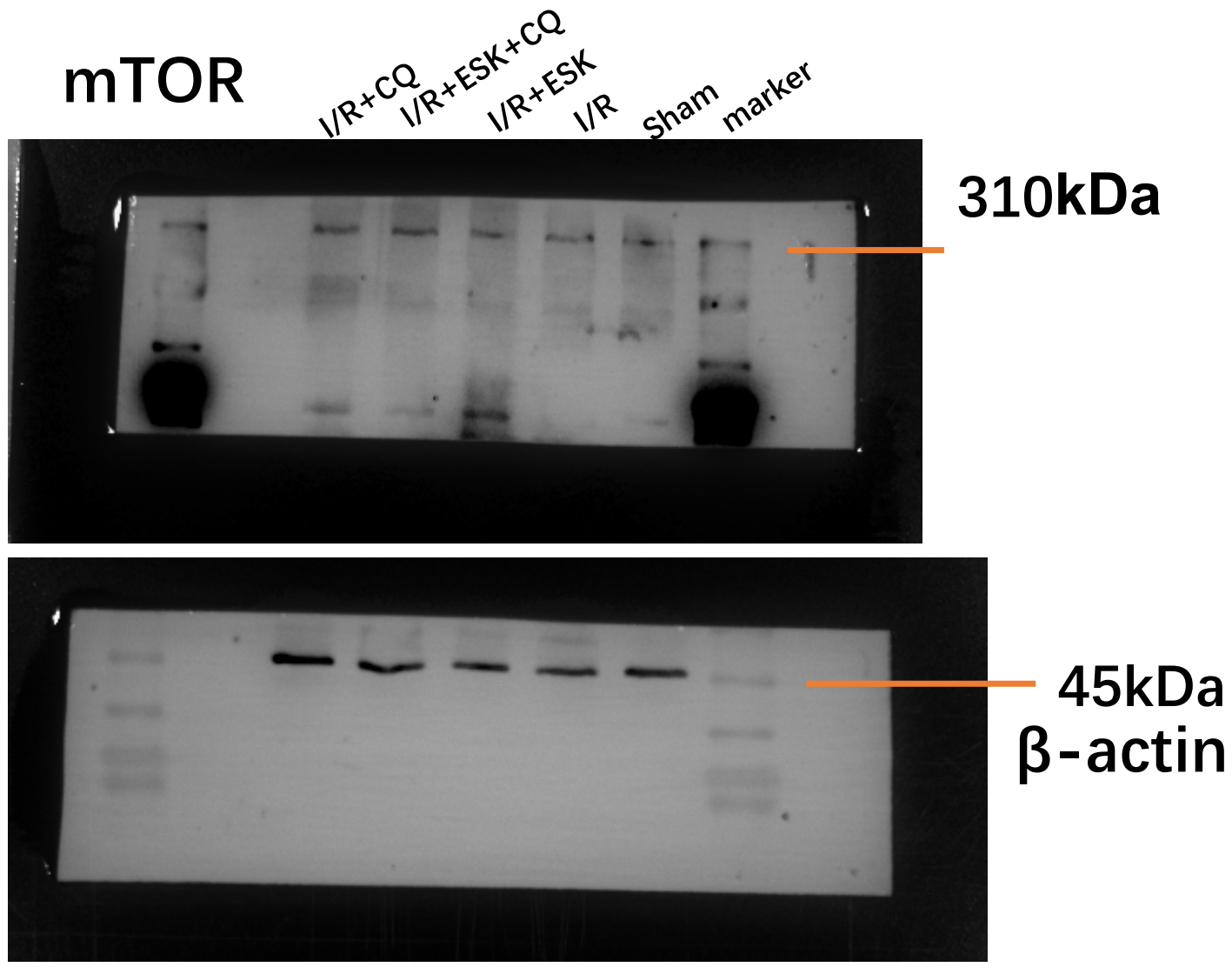
mTOR
I/R+CQ
I/R+ESK+CQ
I/R+ESK
I/R
Sham
marker
310kDa
45kDa
β-actin

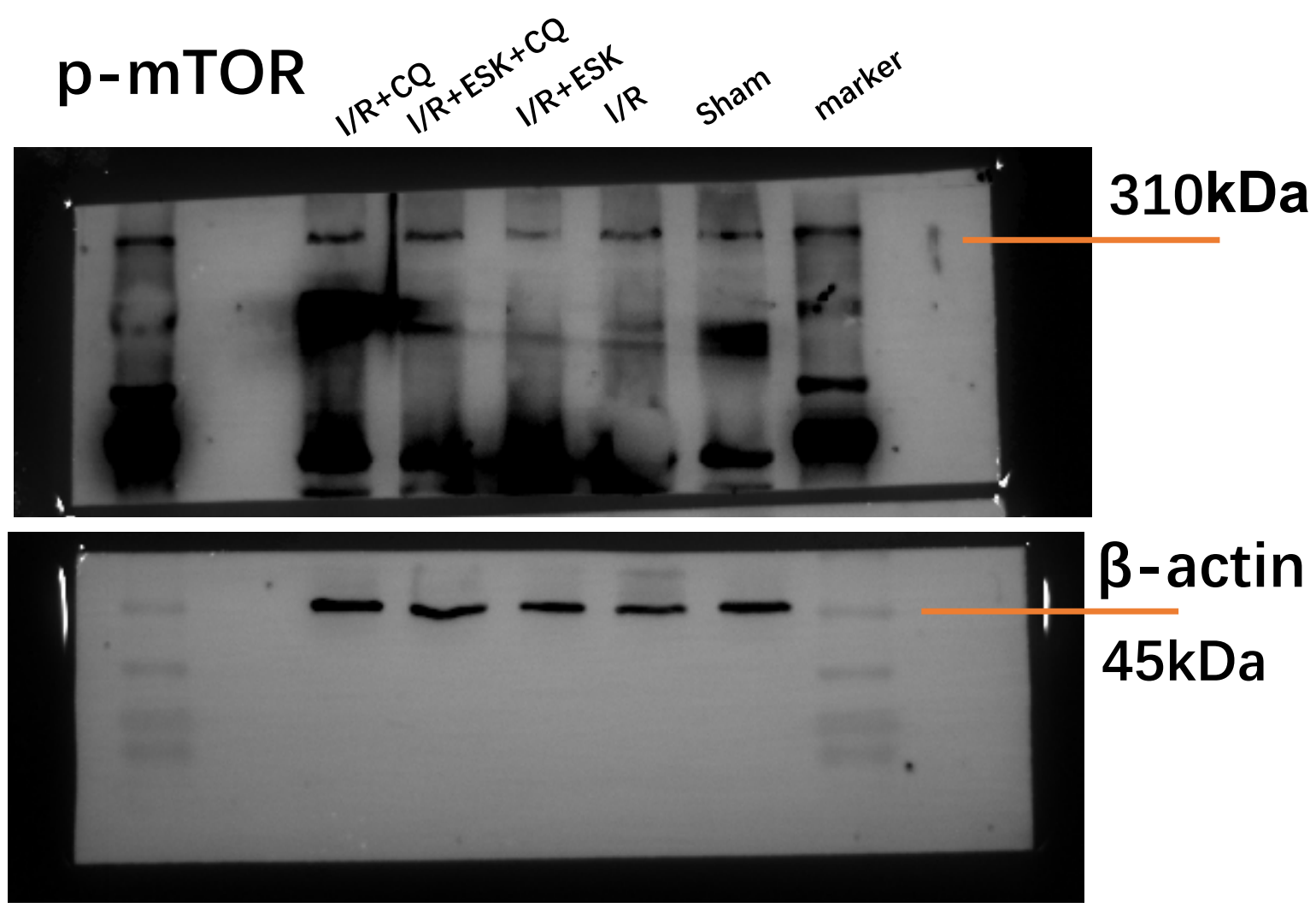
p-mTOR
I/R+CQ
I/R+ESK+CQ
I/R+ESK
I/R
Sham
marker
310kDa
β-actin
45kDa

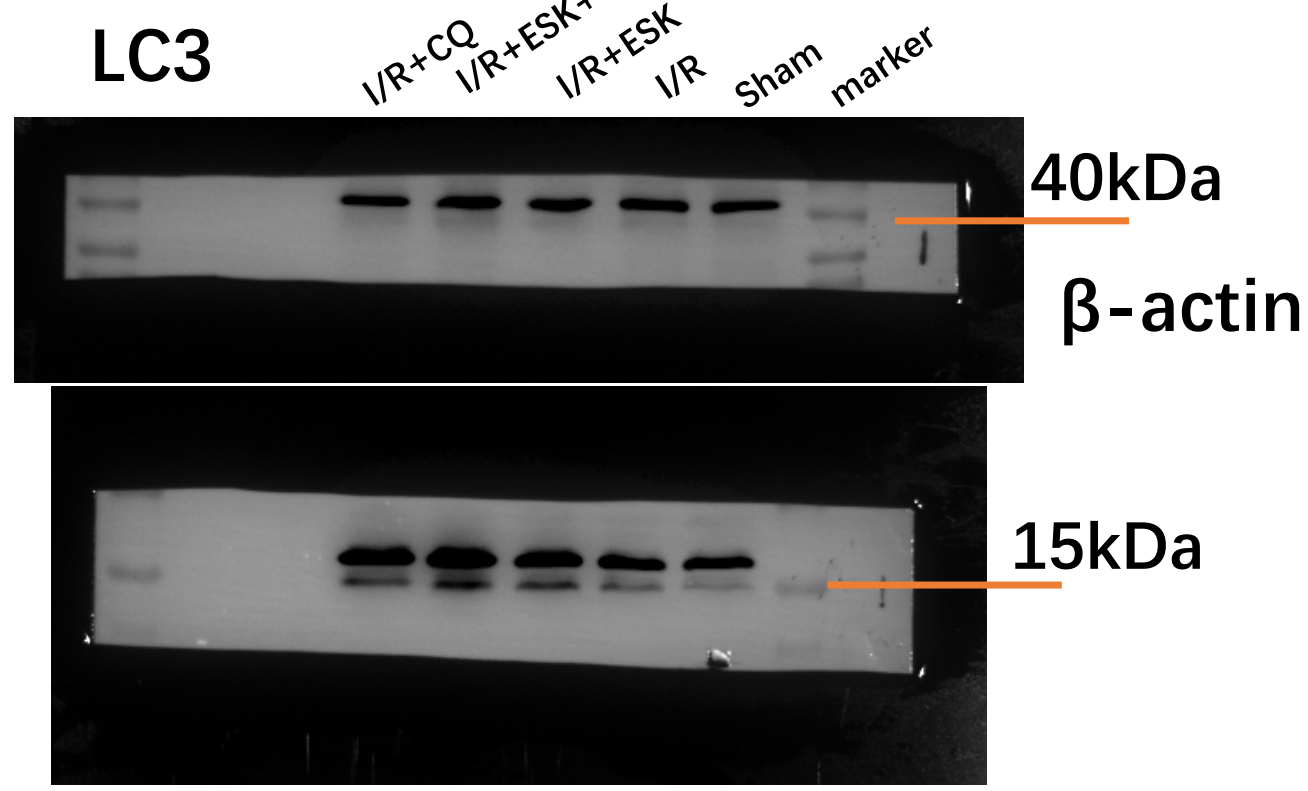
LC3
I/R+CQ
I/R+ESK+CQ
I/R+ESK
I/R
Sham
marker
40kDa
β-actin
15kDa

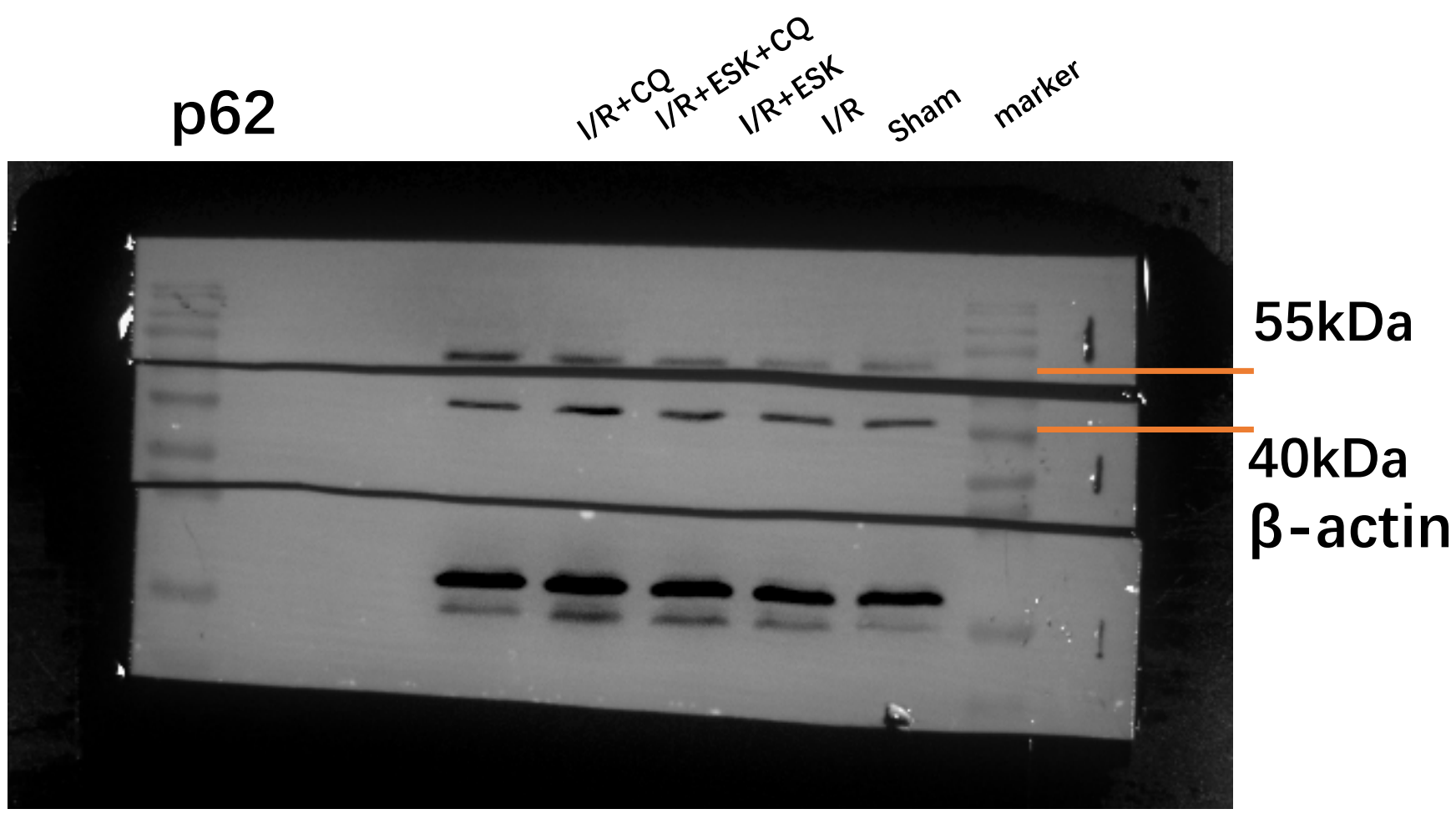
p62
I/R+CQ
I/R+ESK+CQ
I/R+ESK
I/R
Sham
marker
55kDa
40kDa
β-actin

Replicate 5

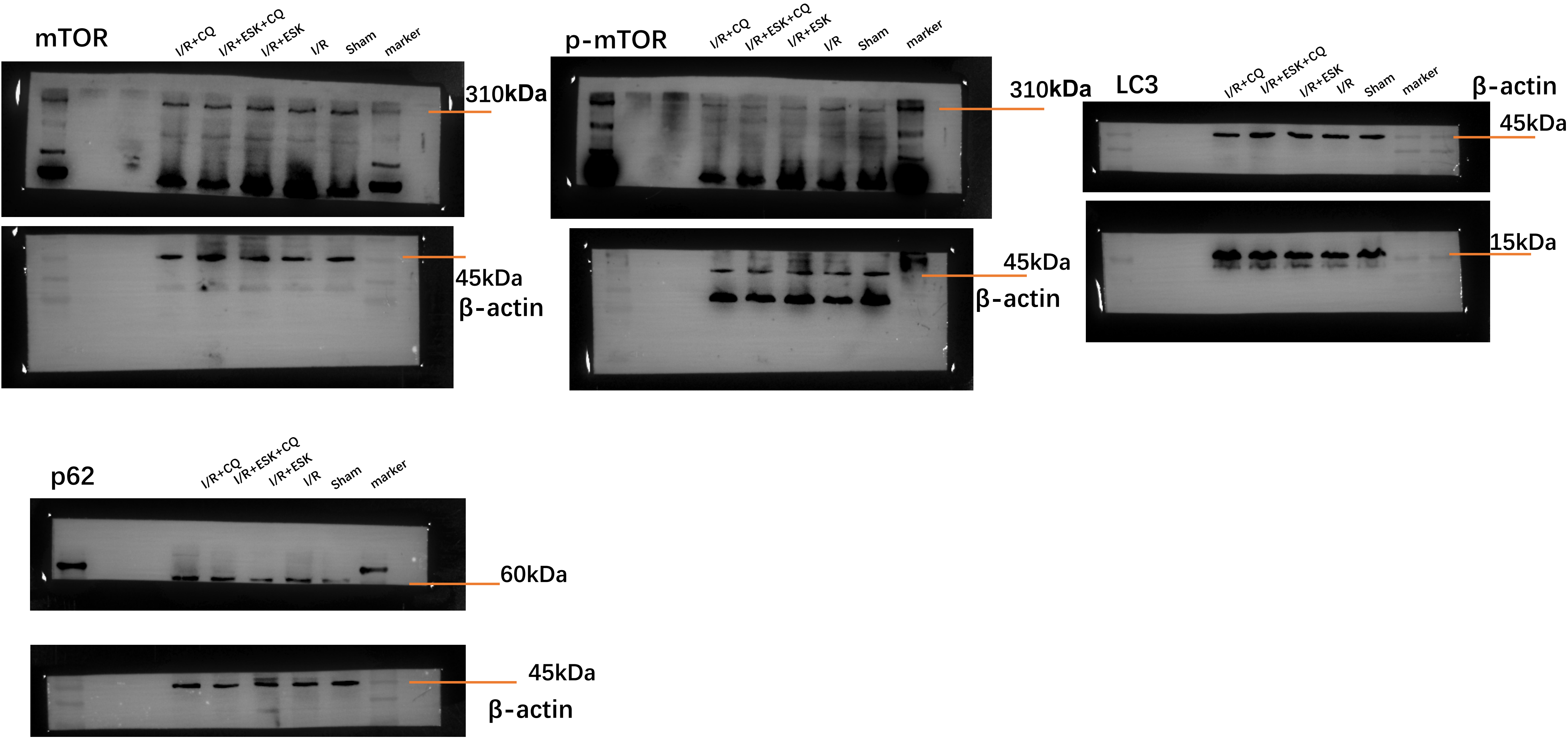

mTOR
I/R+CQ
I/R+ESK+CQ
I/R+ESK
I/R
Sham
marker
310kDa
45kDa
β-actin
p-mTOR
I/R+CQ
I/R+ESK+CQ
I/R+ESK
I/R
Sham
marker
310kDa
45kDa
β-actin
LC3
I/R+CQ
I/R+ESK+CQ
I/R+ESK
I/R
Sham
marker
β-actin
45kDa
15kDa
p62
I/R+CQ
I/R+ESK+CQ
I/R+ESK
I/R
Sham
marker
60kDa
45kDa
β-actin
