# Supplementary material for: Esketamine exerts a protective effect against skeletal muscle injury induced by hindlimb ischemia-reperfusion in mice by regulating autophagy
Source: PLoS One. 2026 Sep 15;21(9):e0358125. doi: 10.1371/journal.pone.0358125 (PMC13577385; doi:10.1371/journal.pone.0358125)
Supplement: Supplementary file 4 [file pone.0358125.s012.docx]

Dear Editor,

We sincerely thank you for your careful evaluation of our manuscript and for giving us the opportunity to revise and improve our work. We greatly appreciate the constructive comments and valuable suggestions provided by you and the reviewers.

In accordance with the journal requirements, we have carefully revised the manuscript format to ensure compliance with the PLOS ONE style and submission guidelines. We have also removed all funding-related information from the manuscript text and added captions for the Supporting Information files at the end of the manuscript. In addition, we have provided the original uncropped and unadjusted images underlying the Western blot results as Supporting Information, in accordance with the journal’s requirements for blot and gel reporting.

We sincerely appreciate the time and effort devoted by the Editor and the reviewers to evaluating our manuscript. Their comments and suggestions have been invaluable in improving the quality, clarity, and rigor of our work.

Our detailed point-by-point responses to the reviewers’ comments are provided below.

**Response to Reviewer #1**

We sincerely thank the reviewer for the careful evaluation of our manuscript and for the detailed comments and annotations provided in the PDF file. We have carefully considered all of the reviewer’s suggestions and revised the manuscript accordingly where appropriate. The major revisions include the following:

• All abbreviated terms have been spelled out at their first occurrence in the manuscript.

• The total number of animals used in the study has been added.

• The concentration of isoflurane used for anesthesia has been specified.

We would also like to provide clarification regarding the following two points:

1. “SYBR”

Thank you for pointing this out. SYBR is used as the name of the fluorescent dye and is not an abbreviation requiring expansion. Therefore, we have retained “SYBR” as written in the manuscript.

2. P values in Results subsection headings

Because each outcome involves five experimental groups, listing all pairwise P values in the headings would make them lengthy and difficult to read. Instead, we have used the compact-letter display in all figures: groups not sharing a letter are significantly different (P < 0.05), and this convention is explained in the figure legends. We believe this is both clear and reader-friendly, and we have retained the descriptive headings. We hope these responses are satisfactory and thank the reviewer again for the helpful feedback.

**Response to Reviewer #2**

We sincerely thank the reviewer for the careful evaluation of our manuscript and for the constructive comments and suggestions. We have carefully considered each comment and revised the manuscript accordingly. Our point-by-point responses are provided below.

Comment 1:

The study only covers the acute period (24 hours after reperfusion). Selecting only an early time point does not allow for the assessment of muscle tissue recovery dynamics, regeneration of damaged fibers, possible normalization of neuromuscular transmission, and long-term functional outcomes. Including additional time points, for example, 7 and 14 days after reperfusion, would allow not only to track the development of esketamine's protective effect over time but also to assess whether the observed improvement is transient or persists over a longer period. Furthermore, long-term analysis would allow for the drug's effect on tissue remodeling, fibrosis, and contractile function restoration. This is a critical point.

Response:

We sincerely thank the reviewer for this valuable comment. We agree that additional observation time points would be meaningful for evaluating the persistence of the protective effect of esketamine and the long-term recovery of skeletal muscle.

However, the primary objective of the present study was to investigate the protective effect of esketamine during the acute phase of skeletal muscle ischemia-reperfusion injury. Therefore, we selected 24 h after reperfusion as the observation time point to evaluate acute tissue injury, inflammatory responses, functional impairment, and autophagy-related molecular changes.

We acknowledge that the present study cannot determine whether the protective effect of esketamine persists into the later stages of muscle repair and remodeling. This limitation was already acknowledged in the original manuscript, and we have carefully avoided extending our conclusions beyond the acute phase of ischemia-reperfusion injury. In addition, we have further clarified in the manuscript that future studies should include later observation time points to investigate skeletal muscle regeneration, fibrosis, neuromuscular recovery, and long-term restoration of contractile function.

We sincerely thank the reviewer again for this important suggestion.

Comment 2:

For Western blotting and real-time PCR, n=3 was used. This is the minimum acceptable sample size for such analyses. This sample size greatly reduces statistical power and makes the study more vulnerable to random outliers. This sample size should be increased (at least to n=5). This is a critical comment.

Response:

We sincerely thank the reviewer for this important comment. We agree that increasing the number of biological replicates can improve the statistical robustness and reliability of the molecular analyses.

In response to the reviewer’s suggestion, we performed additional experiments using an independent animal cohort under the same experimental conditions as those used in the original study. Two additional biological samples were included in each group, thereby increasing the sample size for both Western blot and RT-qPCR analyses from n=3 to n=5 per group.

The corresponding analyses were repeated using the expanded sample size. **The overall trends and main findings were generally consistent with those observed in the original analyses, although minor changes were observed in some statistical comparisons after increasing the sample size.** The relevant Methods, Results, figures, and figure legends have been updated accordingly.

We sincerely thank the reviewer for this valuable suggestion, which has improved the robustness and reliability of our molecular findings.

Comment 3:

In the ESK+CQ group, a heterogeneous gene expression response was observed (like decreased Ccl2, but increased Il1b). The authors attribute this to the complexity and time-dependence of the inflammatory response. However, I believe this could be a consequence of the small sample size (n=3). See above.

Response:

We sincerely thank the reviewer for this insightful comment. We agree that the relatively small sample size in the original RT–qPCR analysis may have made the results more susceptible to inter-individual variability. Following the reviewer’s suggestion, we increased the number of independent biological replicates in each group from n = 3 to n = 5 and repeated the statistical analysis. After incorporating the additional independent biological samples, the overall gene expression trends remained largely consistent with those observed in the original analysis, and the discordant expression changes of Ccl2 and Il1b in the ESK + CQ group persisted.

In addition to inhibiting autophagy, CQ may affect inflammatory mediators through other pharmacological actions. However, the available data remain insufficient to elucidate the biological basis underlying these differences in expression, and the uncertainty associated with the limited sample size cannot be completely excluded. Therefore, in the revised manuscript, we have adopted a more cautious mechanistic interpretation of the RT–qPCR results. We present the pleiotropic pharmacological effects of CQ only as a possible explanation and explicitly state that further studies are required to validate this observation.

We again thank the reviewer for this valuable comment, which has helped us interpret the relevant findings with greater caution and rigor.

Comment 4:

The authors focus on autophagy and inflammation, but do not evaluate the development of mitochondrial dysfunction in skeletal muscles, which plays a crucial role in I/R. Meanwhile, I/R damage is known to be accompanied by impaired mitochondrial respiratory function, decreased calcium retention capacity, and accumulation of lipid peroxidation products (doi:10.1152/ajpcell.00356.2015; doi:10.32604/biocell.2025.061798; doi:10.1007/s12035-017-0503-9). In addition, there is data in the literature on the possible effect of esketamine on oxidative phosphorylation and mitochondrial function in general. Including these aspects in the discussion, or at least acknowledging this limitation, would allow for a more complete characterization of the mechanisms underlying the observed effects and identify promising directions for future research.

Response:

We sincerely thank the reviewer for this valuable comment. We agree that mitochondrial dysfunction is an important pathological mechanism in skeletal muscle ischemia-reperfusion injury.

In the revised Discussion, we have added a description of mitochondrial respiratory dysfunction, impaired calcium retention capacity, and lipid peroxidation during skeletal muscle ischemia-reperfusion injury, together with relevant references. We have also discussed the possible relationship between mitochondrial homeostasis, mitophagy, and the autophagy-related changes observed in the present study.

In the limitations section, we have further clarified that mitochondrial function and mitophagy-related indicators were not directly evaluated in this study. Therefore, whether esketamine protects skeletal muscle by regulating mitochondrial homeostasis remains to be determined in future studies.

We thank the reviewer again for this suggestion, which has helped us provide a more complete and balanced discussion of the possible protective mechanisms of esketamine.

We hope that the revisions and responses provided above have adequately addressed the concerns raised by the reviewers. Thank you for your time and consideration.

Sincerely,
Xiuli Wang
On behalf of all authors
