# Supplementary material for: Esketamine exerts a protective effect against skeletal muscle injury induced by hindlimb ischemia-reperfusion in mice by regulating autophagy
Source: PLoS One. 2026 Sep 15;21(9):e0358125. doi: 10.1371/journal.pone.0358125 (PMC13577385; doi:10.1371/journal.pone.0358125)
Supplement: Peer review history

# Peer review: Decision Letter 0

**DOI:** 10.1371/journal.pone.0358125.r001

**Authors:** Atsushi Asakura

23 Jun 2026

Dear Dr. Wang,

Thank you for submitting your manuscript to PLOS ONE. After careful consideration, we feel that it has merit but does not fully meet PLOS ONE’s publication criteria as it currently stands. Therefore, we invite you to submit a revised version of the manuscript that addresses the points raised during the review process.

Please submit your revised manuscript by Aug 07 2026 11:59PM. If you will need more time than this to complete your revisions, please reply to this message or contact the journal office at plosone@plos.org. When you're ready to submit your revision, log on to https://www.editorialmanager.com/pone/ and select the 'Submissions Needing Revision' folder to locate your manuscript file.

- A letter that responds to each point raised by the academic editor and reviewer(s). You should upload this letter as a separate file labeled 'Response to Reviewers'.
- A marked-up copy of your manuscript that highlights changes made to the original version. You should upload this as a separate file labeled 'Revised Manuscript with Track Changes'.
- An unmarked version of your revised paper without tracked changes. You should upload this as a separate file labeled 'Manuscript'.

If you would like to make changes to your financial disclosure, please include your updated statement in your cover letter. Guidelines for resubmitting your figure files are available below the reviewer comments at the end of this letter.

If applicable, we recommend that you deposit your laboratory protocols in protocols.io to enhance the reproducibility of your results. Protocols.io assigns your protocol its own identifier (DOI) so that it can be cited independently in the future. For instructions see: https://journals.plos.org/plosone/s/submission-guidelines#loc-laboratory-protocols. Additionally, PLOS ONE offers an option for publishing peer-reviewed Lab Protocol articles, which describe protocols hosted on protocols.io. Read more information on sharing protocols at https://plos.org/protocols?utm_medium=editorial-email&utm_source=authorletters&utm_campaign=protocols.

As the corresponding author, your ORCID iD is verified in the submission system and will appear in the published article. PLOS supports the use of ORCID, and we encourage all coauthors to register for an ORCID iD and use it as well. Please encourage your coauthors to verify their ORCID iD within the submission system before final acceptance, as unverified ORCID iDs will not appear in the published article. *Only*  the individual author can complete the verification step; PLOS staff *cannot*  verify ORCID iDs on behalf of authors.

We look forward to receiving your revised manuscript.

Kind regards,

Atsushi Asakura, Ph.D

Academic Editor

PLOS One

Journal Requirements:

When submitting your revision, we need you to address these additional requirements.

1.Please ensure that your manuscript meets PLOS ONE's style requirements, including those for file naming. The PLOS ONE style templates can be found at

https://journals.plos.org/plosone/s/file?id=wjVg/PLOSOne_formatting_sample_main_body.pdf and

https://journals.plos.org/plosone/s/file?id=ba62/PLOSOne_formatting_sample_title_authors_affiliations.pdf

2. Please note that funding information should not appear in any section or other areas of your manuscript. We will only publish funding information present in the Funding Statement section of the online submission form. Please remove any funding-related text from the manuscript.

3. Please include captions for your Supporting Information files at the end of your manuscript, and update any in-text citations to match accordingly. Please see our Supporting Information guidelines for more information: http://journals.plos.org/plosone/s/supporting-information.

4. PLOS ONE now requires that authors provide the original uncropped and unadjusted images underlying all blot or gel results reported in a submission’s figures or Supporting Information files. This policy and the journal’s other requirements for blot/gel reporting and figure preparation are described in detail at https://journals.plos.org/plosone/s/figures#loc-blot-and-gel-reporting-requirements and https://journals.plos.org/plosone/s/figures#loc-preparing-figures-from-image-files. When you submit your revised manuscript, please ensure that your figures adhere fully to these guidelines and provide the original underlying images for all blot or gel data reported in your submission. See the following link for instructions on providing the original image data: https://journals.plos.org/plosone/s/figures#loc-original-images-for-blots-and-gels.

In your cover letter, please note whether your blot/gel image data are in Supporting Information or posted at a public data repository, provide the repository URL if relevant, and provide specific details as to which raw blot/gel images, if any, are not available. Email us at plosone@plos.org if you have any questions.

5. If the reviewer comments include a recommendation to cite specific previously published works, please review and evaluate these publications to determine whether they are relevant and should be cited. There is no requirement to cite these works unless the editor has indicated otherwise.

[Note: HTML markup is below. Please do not edit.]

Reviewers' comments:

Reviewer's Responses to Questions

**Comments to the Author**

1. Is the manuscript technically sound, and do the data support the conclusions?

Reviewer #1: Yes

Reviewer #2: Partly

**********

2. Has the statistical analysis been performed appropriately and rigorously?

Reviewer #1: Yes

Reviewer #2: No

**********

3. Have the authors made all data underlying the findings in their manuscript fully available?

The PLOS Data policy

Reviewer #1: Yes

Reviewer #2: Yes

**********

4. Is the manuscript presented in an intelligible fashion and written in standard English?

Reviewer #1: Yes

Reviewer #2: Yes

**********

Reviewer #1: Minor revision requirieds. Changes and suggestions PDF file. Minor revision requirieds. Changes and suggestions PDF file. Minor revision requirieds. Changes and suggestions PDF file. Minor revision requirieds. Changes and suggestions PDF file.

Reviewer #2: The manuscript by Liu et al addresses the pressing issue of perioperative skeletal muscle protection in tourniquet-associated ischemia-reperfusion (I/R) of the lower extremity. The authors chose esketamine, a well-known analgesic and sedative agent in anesthesiology, as their study agent. In recent years, esketamine has attracted attention for its potential tissue-protective effects in various pathological conditions associated with ischemia, oxidative stress, and inflammation. The study was performed on a mouse model using a comprehensive multiparametric approach, including assessment of regional perfusion using laser speckle imaging, functional tests, histological analysis, measurement of systemic inflammatory cytokines, and molecular biological methods (Western blotting, real-time PCR) to study autophagy and inflammatory signaling pathways. This design allowed the authors to evaluate both the damaging effects of I/R and the protective potential of esketamine. I would consider the thoughtful use of a pharmacological inhibitor of autophagy (chloroquine phosphate) a strength of the study. This allowed the authors not only to document the activation of autophagy by esketamine but also to demonstrate a partial abolition of the protective effect when it was blocked. This confirms the involvement of the autophagic pathway in the protective effect of esketamine. Furthermore, in my opinion, the separate study of contractility during direct muscle stimulation and stimulation via the sciatic nerve is noteworthy. This allowed the authors to identify an important detail related to the predominant effect of esketamine on the muscle component rather than the nerve component in the acute 24 h phase after reperfusion. This level of detail lends additional translational value to the study.

Below are the main comments and recommendations for revision of the manuscript.

1. The study only covers the acute period (24 hours after reperfusion). Selecting only an early time point does not allow for the assessment of muscle tissue recovery dynamics, regeneration of damaged fibers, possible normalization of neuromuscular transmission, and long-term functional outcomes. Including additional time points, for example, 7 and 14 days after reperfusion, would allow not only to track the development of esketamine's protective effect over time but also to assess whether the observed improvement is transient or persists over a longer period. Furthermore, long-term analysis would allow for the drug's effect on tissue remodeling, fibrosis, and contractile function restoration. This is a critical point.

2. For Western blotting and real-time PCR, n=3 was used. This is the minimum acceptable sample size for such analyses. This sample size greatly reduces statistical power and makes the study more vulnerable to random outliers. This sample size should be increased (at least to n=5). This is a critical comment.

3. In the ESK+CQ group, a heterogeneous gene expression response was observed (like decreased Ccl2, but increased Il1b). The authors attribute this to the complexity and time-dependence of the inflammatory response. However, I believe this could be a consequence of the small sample size (n=3). See above.

4. The authors focus on autophagy and inflammation, but do not evaluate the development of mitochondrial dysfunction in skeletal muscles, which plays a crucial role in I/R. Meanwhile, I/R damage is known to be accompanied by impaired mitochondrial respiratory function, decreased calcium retention capacity, and accumulation of lipid peroxidation products (doi:10.1152/ajpcell.00356.2015; doi:10.32604/biocell.2025.061798; doi:10.1007/s12035-017-0503-9). In addition, there is data in the literature on the possible effect of esketamine on oxidative phosphorylation and mitochondrial function in general. Including these aspects in the discussion, or at least acknowledging this limitation, would allow for a more complete characterization of the mechanisms underlying the observed effects and identify promising directions for future research.

**********

what does this mean?). If published, this will include your full peer review and any attached files.

If you choose “no”, your identity will remain anonymous but your review may still be made public.

**Do you want your identity to be public for this peer review?**  For information about this choice, including consent withdrawal, please see our Privacy Policy

Reviewer #1: No

Reviewer #2: No

**********

[NOTE: If reviewer comments were submitted as an attachment file, they will be attached to this email and accessible via the submission site. Please log into your account, locate the manuscript record, and check for the action link "View Attachments". If this link does not appear, there are no attachment files.]

To ensure your figures meet our technical requirements, please review our figure guidelines: https://journals.plos.org/plosone/s/figures

You may also use PLOS’s free figure tool, NAAS, to help you prepare publication quality figures: https://journals.plos.org/plosone/s/figures#loc-tools-for-figure-preparation.

NAAS will assess whether your figures meet our technical requirements by comparing each figure against our figure specifications.

Attachment Submitted filename: PONE-D-26-18635_reviewer.pdf

# Author comment: Author response to Decision Letter 1

**DOI:** 10.1371/journal.pone.0358125.r002

12 Jul 2026

Dear Editor,

We sincerely thank you for your careful evaluation of our manuscript and for giving us the opportunity to revise and improve our work. We greatly appreciate the constructive comments and valuable suggestions provided by you and the reviewers.

In accordance with the journal requirements, we have carefully revised the manuscript format to ensure compliance with the PLOS ONE style and submission guidelines. We have also removed all funding-related information from the manuscript text and added captions for the Supporting Information files at the end of the manuscript. In addition, we have provided the original uncropped and unadjusted images underlying the Western blot results as Supporting Information, in accordance with the journal’s requirements for blot and gel reporting.

We sincerely appreciate the time and effort devoted by the Editor and the reviewers to evaluating our manuscript. Their comments and suggestions have been invaluable in improving the quality, clarity, and rigor of our work.

Our detailed point-by-point responses to the reviewers’ comments are provided below.

Response to Reviewer #1

We sincerely thank the reviewer for the careful evaluation of our manuscript and for the detailed comments and annotations provided in the PDF file. We have carefully considered all of the reviewer’s suggestions and revised the manuscript accordingly where appropriate. The major revisions include the following:

• All abbreviated terms have been spelled out at their first occurrence in the manuscript.

• The total number of animals used in the study has been added.

• The concentration of isoflurane used for anesthesia has been specified.

We would also like to provide clarification regarding the following two points:

1. “SYBR”

Thank you for pointing this out. SYBR is used as the name of the fluorescent dye and is not an abbreviation requiring expansion. Therefore, we have retained “SYBR” as written in the manuscript.

2. P values in Results subsection headings

Because each outcome involves five experimental groups, listing all pairwise P values in the headings would make them lengthy and difficult to read. Instead, we have used the compact-letter display in all figures: groups not sharing a letter are significantly different (P < 0.05), and this convention is explained in the figure legends. We believe this is both clear and reader-friendly, and we have retained the descriptive headings. We hope these responses are satisfactory and thank the reviewer again for the helpful feedback.

Response to Reviewer #2

We sincerely thank the reviewer for the careful evaluation of our manuscript and for the constructive comments and suggestions. We have carefully considered each comment and revised the manuscript accordingly. Our point-by-point responses are provided below.

Comment 1:

The study only covers the acute period (24 hours after reperfusion). Selecting only an early time point does not allow for the assessment of muscle tissue recovery dynamics, regeneration of damaged fibers, possible normalization of neuromuscular transmission, and long-term functional outcomes. Including additional time points, for example, 7 and 14 days after reperfusion, would allow not only to track the development of esketamine's protective effect over time but also to assess whether the observed improvement is transient or persists over a longer period. Furthermore, long-term analysis would allow for the drug's effect on tissue remodeling, fibrosis, and contractile function restoration. This is a critical point.

Response:

We sincerely thank the reviewer for this valuable comment. We agree that additional observation time points would be meaningful for evaluating the persistence of the protective effect of esketamine and the long-term recovery of skeletal muscle.

However, the primary objective of the present study was to investigate the protective effect of esketamine during the acute phase of skeletal muscle ischemia-reperfusion injury. Therefore, we selected 24 h after reperfusion as the observation time point to evaluate acute tissue injury, inflammatory responses, functional impairment, and autophagy-related molecular changes.

We acknowledge that the present study cannot determine whether the protective effect of esketamine persists into the later stages of muscle repair and remodeling. This limitation was already acknowledged in the original manuscript, and we have carefully avoided extending our conclusions beyond the acute phase of ischemia-reperfusion injury. In addition, we have further clarified in the manuscript that future studies should include later observation time points to investigate skeletal muscle regeneration, fibrosis, neuromuscular recovery, and long-term restoration of contractile function.

We sincerely thank the reviewer again for this important suggestion.

Comment 2:

For Western blotting and real-time PCR, n=3 was used. This is the minimum acceptable sample size for such analyses. This sample size greatly reduces statistical power and makes the study more vulnerable to random outliers. This sample size should be increased (at least to n=5). This is a critical comment.

Response:

We sincerely thank the reviewer for this important comment. We agree that increasing the number of biological replicates can improve the statistical robustness and reliability of the molecular analyses.

In response to the reviewer’s suggestion, we performed additional experiments using an independent animal cohort under the same experimental conditions as those used in the original study. Two additional biological samples were included in each group, thereby increasing the sample size for both Western blot and RT-qPCR analyses from n=3 to n=5 per group.

The corresponding analyses were repeated using the expanded sample size. The overall trends and main findings were generally consistent with those observed in the original analyses, although minor changes were observed in some statistical comparisons after increasing the sample size. The relevant Methods, Results, figures, and figure legends have been updated accordingly.

We sincerely thank the reviewer for this valuable suggestion, which has improved the robustness and reliability of our molecular findings.

Comment 3:

In the ESK+CQ group, a heterogeneous gene expression response was observed (like decreased Ccl2, but increased Il1b). The authors attribute this to the complexity and time-dependence of the inflammatory response. However, I believe this could be a consequence of the small sample size (n=3). See above.

Response:

We sincerely thank the reviewer for this insightful comment. We agree that the relatively small sample size in the original RT–qPCR analysis may have made the results more susceptible to inter-individual variability. Following the reviewer’s suggestion, we increased the number of independent biological replicates in each group from n = 3 to n = 5 and repeated the statistical analysis. After incorporating the additional independent biological samples, the overall gene expression trends remained largely consistent with those observed in the original analysis, and the discordant expression changes of Ccl2 and Il1b in the ESK + CQ group persisted.

In addition to inhibiting autophagy, CQ may affect inflammatory mediators through other pharmacological actions. However, the available data remain insufficient to elucidate the biological basis underlying these differences in expression, and the uncertainty associated with the limited sample size cannot be completely excluded. Therefore, in the revised manuscript, we have adopted a more cautious mechanistic interpretation of the RT–qPCR results. We present the pleiotropic pharmacological effects of CQ only as a possible explanation and explicitly state that further studies are required to validate this observation.

We again thank the reviewer for this valuable comment, which has helped us interpret the relevant findings with greater caution and rigor.

Comment 4:

The authors focus on autophagy and inflammation, but do not evaluate the development of mitochondrial dysfunction in skeletal muscles, which plays a crucial role in I/R. Meanwhile, I/R damage is known to be accompanied by impaired mitochondrial respiratory function, decreased calcium retention capacity, and accumulation of lipid peroxidation products (doi:10.1152/ajpcell.00356.2015; doi:10.32604/biocell.2025.061798; doi:10.1007/s12035-017-0503-9). In addition, there is data in the literature on the possible effect of esketamine on oxidative phosphorylation and mitochondrial function in general. Including these aspects in the discussion, or at least acknowledging this limitation, would allow for a more complete characterization of the mechanisms underlying the observed effects and identify promising directions for future research.

Response:

We sincerely thank the reviewer for this valuable comment. We agree that mitochondrial dysfunction is an important pathological mechanism in skeletal muscle ischemia-reperfusion injury.

In the revised Discussion, we have added a description of mitochondrial respiratory dysfunction, impaired calcium retention capacity, and lipid peroxidation during skeletal muscle ischemia-reperfusion injury, together with relevant references. We have also discussed the possible relationship between mitochondrial homeostasis, mitophagy, and the autophagy-related changes observed in the present study.

In the limitations section, we have further clarified that mitochondrial function and mitophagy-related indicators were not directly evaluated in this study. Therefore, whether esketamine protects skeletal muscle by regulating mitochondrial homeostasis remains to be determined in future studies.

We thank the reviewer again for this suggestion, which has helped us provide a more complete and balanced discussion of the possible protective mechanisms of esketamine.

We hope that the revisions and responses provided above have adequately addressed the concerns raised by the reviewers. Thank you for your time and consideration.

Sincerely,

Xiuli Wang

On behalf of all authors

Attachment Submitted filename: Response to Reviewers.docx

# Peer review: Decision Letter 1

**DOI:** 10.1371/journal.pone.0358125.r003

**Authors:** Atsushi Asakura

28 Aug 2026

<p>Esketamine exerts a protective effect against skeletal muscle injury induced by hindlimb ischemia-reperfusion in mice by regulating autophagy.

PONE-D-26-18635R1

Dear Dr. Wang,

We’re pleased to inform you that your manuscript has been judged scientifically suitable for publication and will be formally accepted for publication once it meets all outstanding technical requirements.

Within one week, you’ll receive an e-mail detailing the required amendments. When these have been addressed, you’ll receive a formal acceptance letter and your manuscript will be scheduled for publication.

An invoice will be generated when your article is formally accepted. Please note, if your institution has a publishing partnership with PLOS and your article meets the relevant criteria, all or part of your publication costs will be covered. Please make sure your user information is up-to-date by logging into Editorial Manager at Editorial Manager® and clicking the ‘Update My Information' link at the top of the page. For questions related to billing, please contact billing support.

If your institution or institutions have a press office, please notify them about your upcoming paper to help maximize its impact. If they’ll be preparing press materials, please inform our press team as soon as possible -- no later than 48 hours after receiving the formal acceptance. Your manuscript will remain under strict press embargo until 2 pm Eastern Time on the date of publication. For more information, please contact onepress@plos.org.

Kind regards,

Atsushi Asakura, Ph.D

Academic Editor

PLOS One

Additional Editor Comments (optional):

Reviewers' comments:

Reviewer's Responses to Questions

**Comments to the Author**

Reviewer #1: (No Response)

Reviewer #2: All comments have been addressed

**********

2. Is the manuscript technically sound, and do the data support the conclusions?

Reviewer #1: (No Response)

Reviewer #2: Yes

**********

3. Has the statistical analysis been performed appropriately and rigorously?

Reviewer #1: (No Response)

Reviewer #2: Yes

**********

4. Have the authors made all data underlying the findings in their manuscript fully available?

The PLOS Data policy

Reviewer #1: (No Response)

Reviewer #2: Yes

**********

5. Is the manuscript presented in an intelligible fashion and written in standard English?

Reviewer #1: (No Response)

Reviewer #2: Yes

**********

Reviewer #1: Accept submission. Accept submission. Accept submission. Accept submission. Accept submission.

Reviewer #2: The authors conducted additional experiments, increasing n from 3 to 5 for Western blotting and RT-PCR, which significantly increased the statistical reliability of the results. The main trends remained, confirming the original conclusions. The discussion was also strengthened, the authors added a section on mitochondrial dysfunction and mitophagy, and the limitations section was made more transparent. Responses to all comments were well-reasoned, and the text was edited appropriately. The manuscript has been significantly improved. I recommend acceptance.

**********

what does this mean?). If published, this will include your full peer review and any attached files.

If you choose “no”, your identity will remain anonymous but your review may still be made public.

**Do you want your identity to be public for this peer review?**  For information about this choice, including consent withdrawal, please see our Privacy Policy

Reviewer #1: No

Reviewer #2: No

**********

# Editor report: Acceptance letter

**DOI:** 10.1371/journal.pone.0358125.r004

**Authors:** Atsushi Asakura

PONE-D-26-18635R1

PLOS One

Dear Dr. Wang,

I'm pleased to inform you that your manuscript has been deemed suitable for publication in PLOS One. Congratulations! Your manuscript is now being handed over to our production team.

At this stage, our production department will prepare your paper for publication. This includes ensuring the following:

* All references, tables, and figures are properly cited

* All relevant supporting information is included in the manuscript submission,

* There are no issues that prevent the paper from being properly typeset

You will receive further instructions from the production team, including instructions on how to review your proof when it is ready. Please keep in mind that we are working through a large volume of accepted articles, so please give us a few days to review your paper and let you know the next and final steps.

Lastly, if your institution or institutions have a press office, please let them know about your upcoming paper now to help maximize its impact. If they'll be preparing press materials, please inform our press team within the next 48 hours. Your manuscript will remain under strict press embargo until 2 pm Eastern Time on the date of publication. For more information, please contact onepress@plos.org.

You will receive an invoice from PLOS for your publication fee after your manuscript has reached the completed accept phase. If you receive an email requesting payment before acceptance or for any other service, this may be a phishing scheme. Learn how to identify phishing emails and protect your accounts at https://explore.plos.org/phishing.

If we can help with anything else, please email us at customercare@plos.org.

Thank you for submitting your work to PLOS One and supporting open access.

Kind regards,

PLOS One Editorial Office Staff

on behalf of

Dr. Atsushi Asakura

Academic Editor

PLOS One
